# Heme oxygenase 1 overexpression induces immune evasion of acute myeloid leukemia against natural killer cells by inhibiting CD48

**DOI:** 10.1186/s12967-022-03589-z

**Published:** 2022-09-04

**Authors:** Tianzhuo Zhang, Qin Fang, Ping Liu, Ping Wang, Cheng Feng, Jishi Wang

**Affiliations:** 1 Department of Clinical Medical School, Guizhou Medical University, Guiyang, 550004 China; 2 Department of Hematology, Affiliated Hospital of Guizhou Medical University, Guiyang, 550004 China; 3 Department of Guizhou Province Hematopoietic Stem Cell Transplantation Center and Key Laboratory of Hematological Disease Diagnostic and Treatment Centre, Guiyang, 550004 China; 4 Department of Pharmacy, Affiliated Hospital of Guizhou Medical University, Guiyang, 550004 China

**Keywords:** Acute myeloid leukemia, Heme oxygenase 1, Natural killer cells, CD48, Sirt1

## Abstract

**Background:**

Acute myeloid leukemia (AML) is the most common type of acute leukemia in adults. Given the high relapse rate, more effective treatments are needed to improve clinical outcomes. We previously demonstrated that heme oxygenase 1 (HO1) is overexpressed in AML, while the functional roles of HO1 remain unclear.

**Methods:**

Bioinformatics analysis and flow cytometry were conducted to assess the association between HO1 levels and immune cells or immune checkpoint/ligand molecules in AML patients. Primary natural killer (NK) cells were purified and subsequently co-cultured in vitro with transduced AML cells to determine the effects of HO1 expression on NK cell functions. AML mice models were established to investigate the effects of HO1 expression on cytotoxic effects of NK cells in vivo. The molecular mechanism was studied by flow cytometry, quantitative real-time PCR (qRT-PCR), western blotting, and immunoprecipitation.

**Results:**

Bioinformatics analysis indicated a correlation between HO1 expression and the AML immune microenvironment. The present study findings indicated that HO1 specifically downregulates the expression of CD48, a ligand of the NK cell-activating receptor 2B4, thus decreasing the cytotoxic effect of NK cells. HO1 overexpression promoted tumor growth and inhibited the cytotoxic effect of NK cells in the AML mice model. Mechanistic investigations found that HO1 directly interacted with Sirt1 and increased its expression and deacetylase activity. With the overexpression of HO1, increased Sirt1 in AML cells enabled histone H3K27 deacetylation to suppress CD48 transcription and expression. Administration of Sirt1 inhibitor restored the expression of CD48.

**Conclusions:**

Collectively, HO1 promotes NK cell dysfunction in AML. Therefore, restoring NK cell function by inhibiting HO1 activity is a potential immunotherapeutic approach against AML.

**Supplementary Information:**

The online version contains supplementary material available at 10.1186/s12967-022-03589-z.

## Introduction

Acute myeloid leukemia (AML) is well-established as the most prevalent acute leukemia type in adults [1]. Although new drugs such as venetoclax, gemtuzumab, midostaurin, and enasidenib have recently been approved, the curative rates of AML did not significantly increase, especially for patients at high risk of the disease or who undergo AML relapse following primary therapy [2–4]. AML treatment is often challenging due to disease heterogeneity, the absence of target antigens, and the presence of leukemic stem cells [5, 6]. Immunotherapy targeting immune checkpoint (IC) pathways, including CTLA4, PD1, and PDL1, has recently attracted great attention in cancer therapy because of fewer side effects and greater effects even in metastasis settings [7, 8]. However, the clinical efficacy is limited to a small portion of the patients [9, 10]. Therefore, more effective treatments are emergently needed to improve the clinical outcome.

We previously demonstrated that heme oxygenase 1 (HO1) plays a crucial role in the chemoresistance of AML, and silencing HO1 prolonged the survival of xenograft mouse models [11, 12]. HO1 is a stress-inducible, cytoprotective, and antioxidative enzyme that catalyzes heme degradation into carbon monoxide (CO), biliverdin, and iron. HO1-produced CO is well-established as a major immunomodulatory and immunosuppressive mediator. An increasing body of evidence suggests that HO1 has protumorigenic characteristics [13, 14], as its induction leads to chemoresistance in various human and mouse cancer cell lines [15, 16]. Moreover, in recent studies, HO1 has been shown to mediate M1/M2 macrophage polarization to a certain extent, and myeloid cell lineage-specific knockout of HO1 induces antitumor immunity through tumor-myeloid reprogramming and cytotoxic T cell activation. The role of HO1 as a novel immune checkpoint molecule in myeloid tumor cells has been demonstrated both in mouse tumor models and human samples [17–20]. However, little is known about the immunological roles of HO1 and the significance of targeting HO1 in the treatment of AML. In this study, we sought to elucidate both tumor biological and immunological roles of HO1 in AML using AML patient-derived specimens, human AML cell lines, and mouse AML tumor models and assessed the prospects of targeting HO1 in the treatment of AML.

## Materials and methods

The complete experimental protocols are presented in Additional file 1.

## Results

### Overexpression of HO1 downregulates the level of NK cells in AML patients

**Fig. 1:**
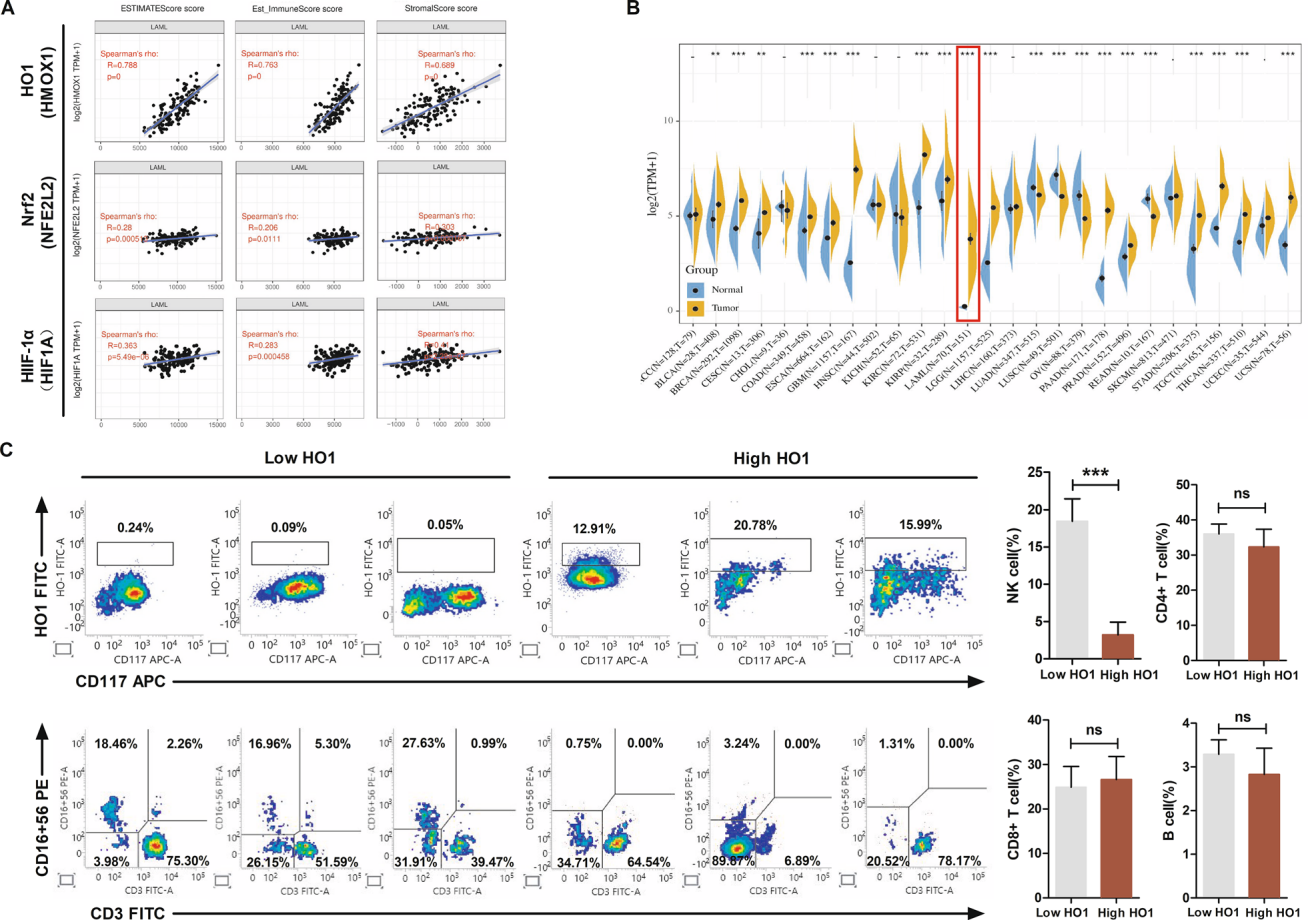
Overexpression of HO1 decreases the levels of NK cells in AML patients. **A** Correlation between HO1, Nrf2, or HIF-1α expression and ESTIMATE, immune, and stromal scores. **B** HO1 levels in 27 types of human cancer. **C** Percentage of NK cells (CD3− CD16+ CD56+) as determined in HO1 high/low AML specimens by the TBNK assay. **p < 0.01, ***p < 0.001 were calculated using the Student's t-test. ns, no significance

Overwhelming evidence substantiates the involvement of HO1 in the generation of a favorable microenvironment, promoting angiogenesis and immune escape in several types of cancers [21, 22]. The relationships between HO1 expression and tumor immune microenvironment was analyzed via the Tumor Immune Estimation Resource (TIMER) database. Stromal, immune, and ESTIMATE (Estimation of STromal and Immune cells in Malignant Tumor tissues with Expression data) scores have huge potential as predictors of the efficacy of immunotherapy [23, 24]. In the present study, a significant positive correlation was found between HO1 gene levels and: ESTIMATE (r = 0.788), immune (r = 0.763), as well as stromal (r = 0.689) scores in acute myeloid leukemia (LAML). In contrast, expression levels of other main antioxidant stress factors, such as Nrf2 and HIF-1α, showed no significant correlation with these scores (Fig. 1A). These findings imply that HO1 expression is correlated with the immune microenvironment of AML. And targeting HO1 is a potential immunotherapy option for AML. Moreover, RNA-seq data analysis in The Cancer Genome Atlas (TCGA) and Genotype-Tissue Expression (GTEx) databases showed that HO1 expression was significantly higher in AML than in adjacent normal tissues (Fig. 1B).

Next, flow cytometry was conducted to assess the association between HO1 levels with four main immune cells (CD4+ T, B, CD8+ T, and NK cells) in AML patients to validate the results from TIMER and TCGA databases above. Patients were assigned to two groups based on HO1 expression levels based on flow cytometry results. The TBNK assay was then conducted to determine the percentage of NK cells in the two groups of patients. The number of NK cells in the HO1-high group was significantly low relative to the HO1-low group. Further analysis showed that HO1 expression was not associated with other immune cells in AML patients (Fig. 1C, Additional file 1: Fig. S1). These findings imply that elevated HO1 levels in AML patients are associated with decreased NK cell levels.

### Overexpression of HO1 inhibited CD48 in AML patients

**Fig. 2:**
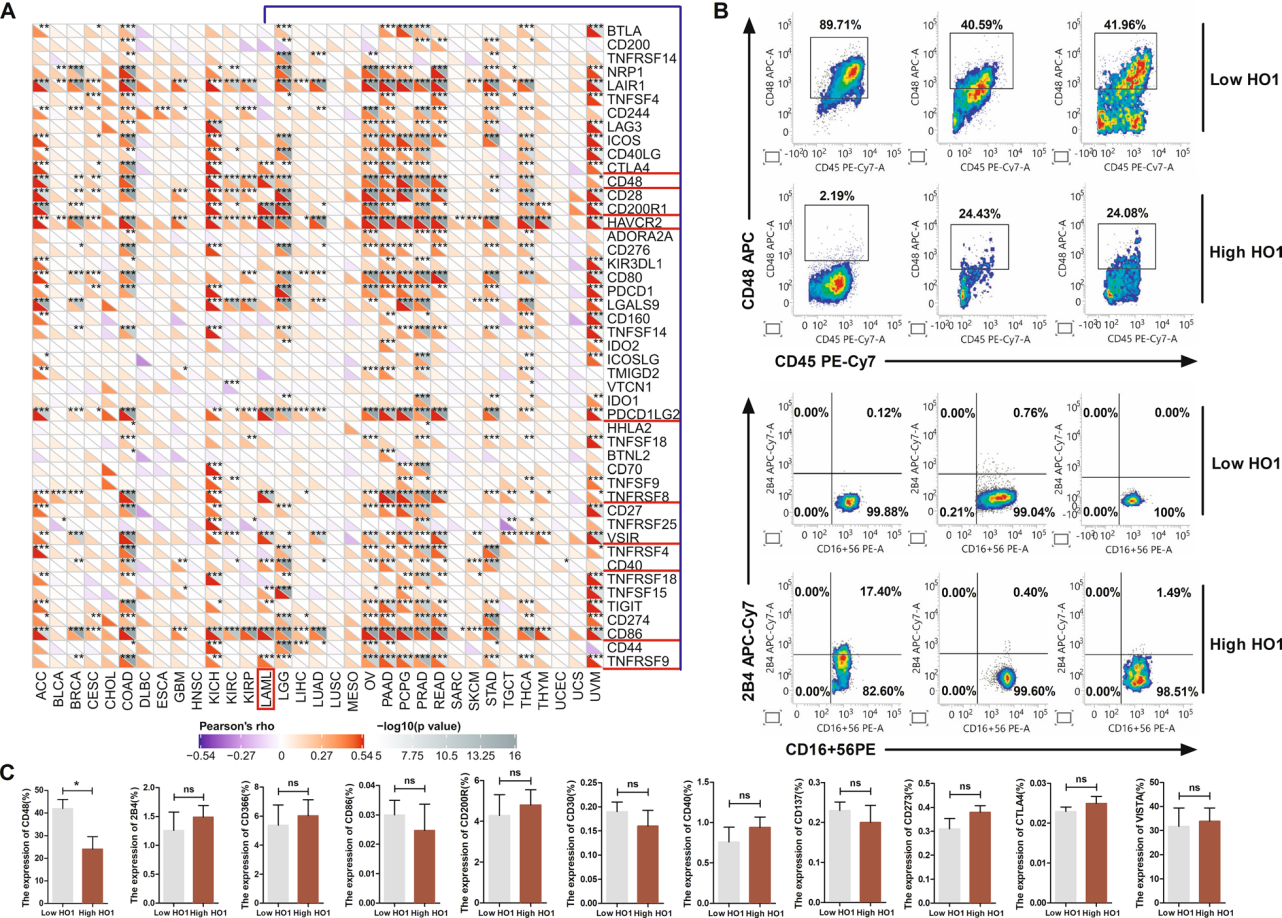
Overexpression of HO1 inhibited CD48 in AML patients. **A** Heatmaps showing the relationship between HO1 expression and 47 immune checkpoint/ligand genes in 33 tumors. **B** CD48 and 2B4 expression as determined in HO1 high/low AML specimens by flow cytometry. **C** Correlation between HO1 and 10 immune checkpoint/ligand molecules. Statistical differences were determined using the Student’s t-test. *p < 0.05, **p < 0.01, ***p < 0.001. *ns* no significance

Studies have proven that immune checkpoint/ligand genes have a great influence on immune cell infiltration and immunotherapy [25]. And current evidence suggests a negative association between HO1 expression and NK cell levels in AML. To explain the underlying mechanism, we explored the associations between HO1 expression and immune checkpoint/ligand genes in human cancers in TCGA database. The results showed that HO1 levels were correlated with several stimulatory checkpoint molecules in a disease-specific pattern in AML. Among all the associations, HO1 expression was significantly correlated with CTLA4, CD48, CD200R1 (CD200R), HAVCR2 (CD366), PDCD1LG2 (CD273), TNFRSF8 (CD30), VSIR (VISTA), CD40, CD86 and TNFRSF9 (CD137) in AML (Fig. 2A). Then flow cytometry was performed to confirm the findings from TCGA analysis. The HO1-high group exhibited suppressed CD48 levels in tumor cells relative to the HO1-low group. Notably, HO1 expression was not correlated with other candidate molecules above. It has been established that 2B4 functions as an activating receptor in NK cells by recognizing its ligand, CD48 [26–28]. However, HO1 expression was not correlated with 2B4, which is inconsistent with the change in CD48 levels (Fig. 2B, C, Additional file 1: Figs. S2, S3). Taken together, these data suggest that HO1 has a negative regulatory effect on the CD48-2B4 axis.

### HO1 is overexpressed in relapsed AML patients

**Fig. 3:**
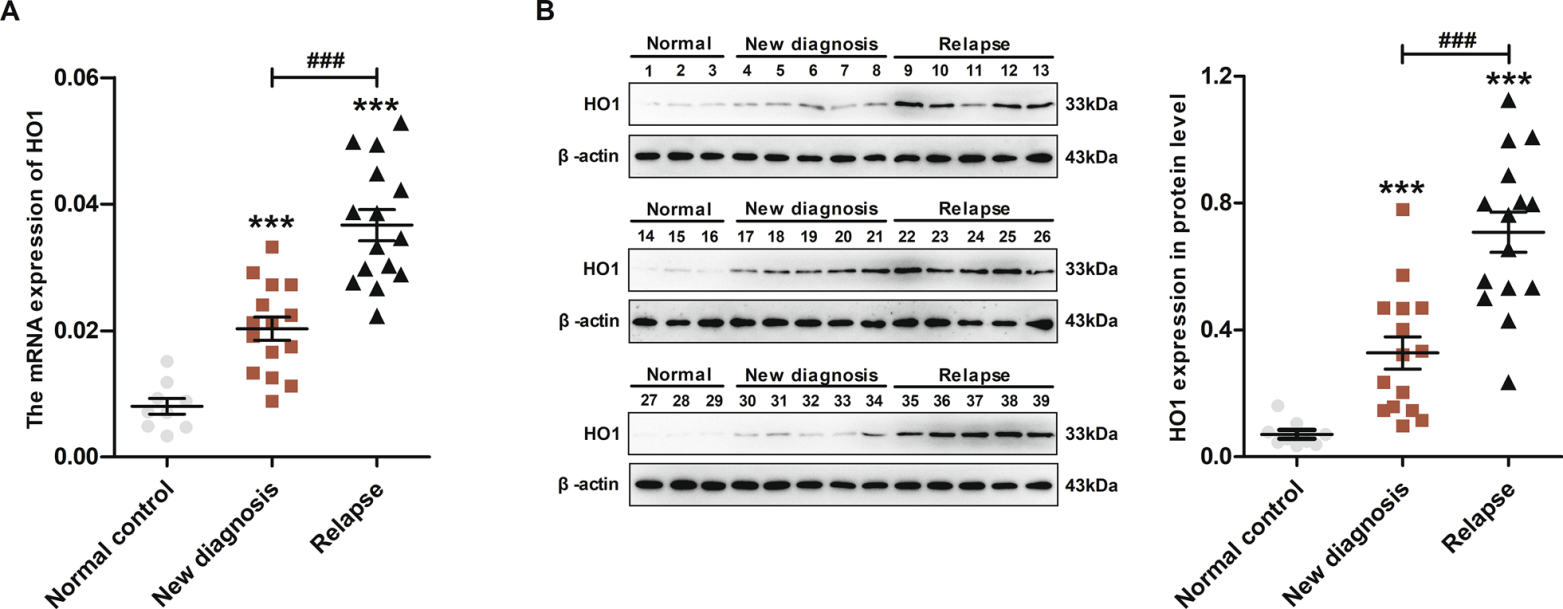
HO1 is overexpressed in relapsed AML patients. **A** HO1 mRNA levels in AML patients (newly diagnosed and relapsed AML). **B** HO1 protein levels in AML patients (newly diagnosed and relapsed AML). ***p < 0.001, and ^###^p < 0.001 were calculated using the Student’s t-test

Protein expression and mRNA levels of HO1 were determined by western blotting and qRT-PCR analyses to determine the expression profile of HO1 in bone marrow mononuclear cells (BMNCs) of AML patients. The HO1 protein and mRNA levels in cells from relapsed AML patients were significantly higher than in newly diagnosed AML patients. Notably, normal donor cells exhibited low expression of HO1 (Fig. 3).

### Overexpression of HO1 in AML cells inhibited NK cell cytotoxicity via targeting the CD48-2B4 axis

**Fig. 4:**
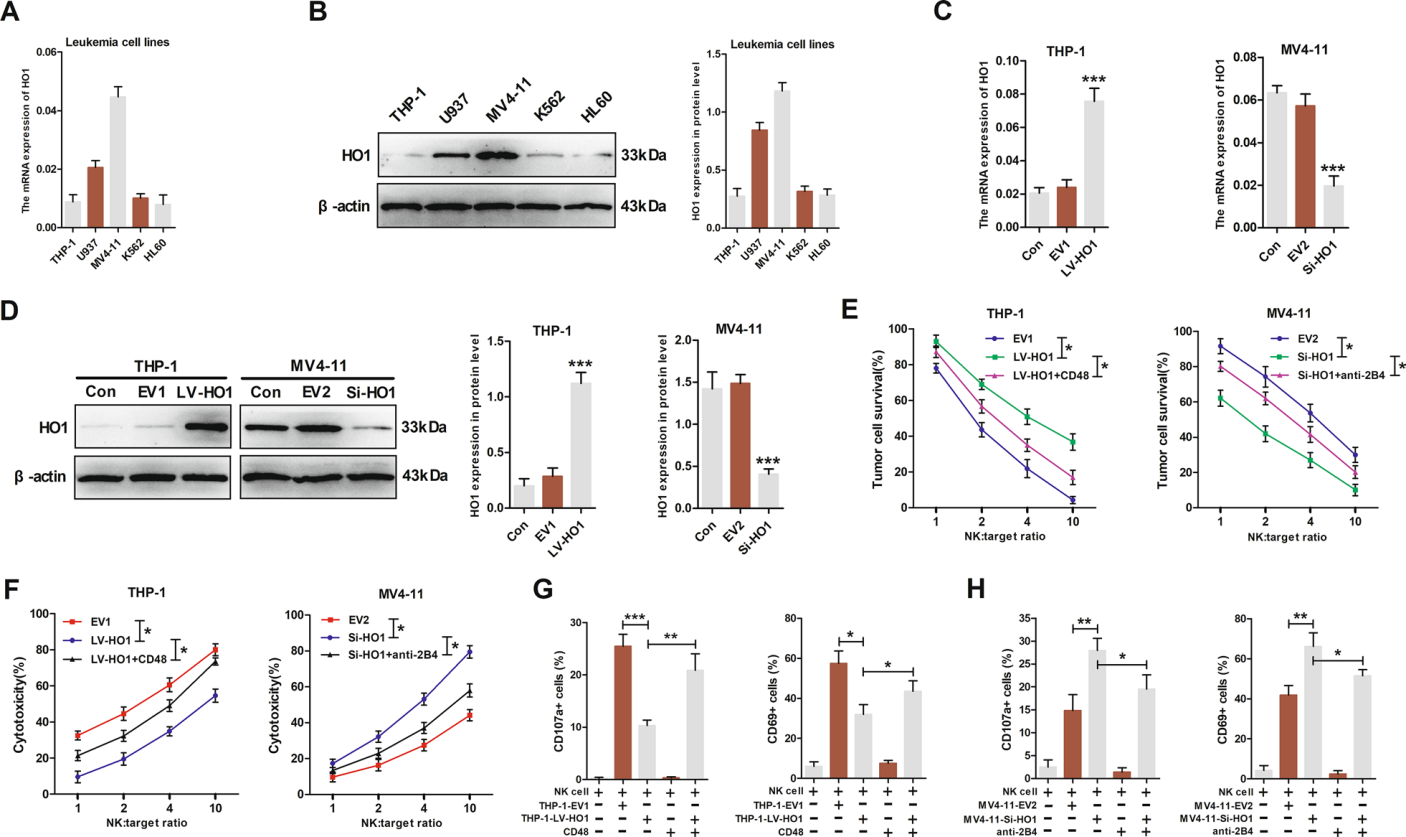
Overexpression of HO1 in AML cells inhibited NK cell cytotoxicity via targeting the CD48-2B4 axis. **A**, **B** HO1 mRNA and protein expression levels in leukemia cell lines. **C**, **D** HO1 was overexpressed in THP-1 and silenced in MV4-11 cell lines. **E** Left graph: percentage survival of Luc‐labeled transduced THP-1 cells after 2 h co-culture with primary NK cells in the presence or absence of CD48 protein, as measured by the bioluminescent imaging system. Right graph: percentage survival of Luc‐labeled transduced MV4-11 cells after 2 h co-culture with primary NK cells in the presence or absence of anti-2B4 antibody, as measured by the bioluminescent imaging system. **F** Primary NK cells (as effector cells) incubated with transduced AML cells (as target cells) at various effector cell/target cell ratios (E:T = 1:1, 2:1, 4:1, 10:1). NK cell-mediated cytotoxicity as determined by LDH release assay. **G** CD107a or CD69 expression in NK cells after 6 h co-culture of NK cells with transduced THP-1 cells (1:1 E:T ratio) in the presence or absence of CD48 protein. **H** Expression of CD107a or CD69 in NK cells after 6 h co-culture of NK cells with transduced MV4-11 cells (1:1 E:T ratio) in the presence or absence of anti-2B4 antibody. *p < 0.05, **p < 0.01, ***p < 0.001 were calculated using Student’s t-test. All experiments were performed in triplicates

Further analysis of the HO1 expression profile in leukemia cell lines was conducted to explore whether the expression of HO1 affects NK cell cytotoxicity. HO1 mRNA and protein levels were elevated in MV4-11 cells relative to other cell lines, whereas THP-1 expression was the lowest in standard AML (non M3) cell lines (Fig. 4A, B). Then transfection of cells with HO1 overexpression lentivirus (LV-HO1) upregulated HO1 expression in THP-1, whereas transfection of MV4-11 with HO1 silence lentivirus LV-HO1-RNAi (Si-HO1) downregulated HO1 expression. The transfection ratio was verified by western blotting and qRT-PCR (Fig. 4C, D).

An increasing body of evidence indicates that NK cells play an important role in AML [29–31]. Next, we evaluated whether HO1 expression affects the survival of transduced AML cells under the cytotoxic effects of NK cells (Fig. 4E, Additional file 1: Fig. S5A, B). Our findings indicated that cells overexpressing HO1 exhibited a significantly higher survival rate than cells with the empty vector. Notably, the knockdown of HO1 decreased the survival rate of transduced cells. The addition of CD48 significantly decreased the survival of THP-1 cells expressing LV-HO1 by restoring the cytotoxic activity of NK cells to a certain extent (Fig. 4E, left graph). Similarly, blocking 2B4 significantly increased the survival rate of MV4-11 cells expressing Si-HO1 by weakening the cytotoxic activity of NK cells to a certain extent (Fig. 4E, right graph).

Furthermore, NK cytotoxicity assays were performed to explore whether HO1 affects the cytotoxic ability of NK cells against the transduced AML cells and the underlying mechanisms (Fig. 4F). These experiments demonstrated significantly reduced cytotoxic activity of cells overexpressing HO1 compared to cells expressing an empty vector, while knockdown of HO1 sensitized the transduced AML cells to NK cells. The addition of CD48 significantly increased the killing of THP-1 cells expressing LV-HO1 by restoring the cytotoxic activity of NK cells to a certain extent (Fig. 4F, left graph). Consistently, blocking 2B4 could significantly reduce cytotoxic activity against MV4-11 cells expressing Si-HO1 by reducing the cytotoxic activity of NK cells to some extent (Fig. 4F, right graph).

On the other hand, HO1 in AML cells significantly affected NK cell activation and degranulation, as shown by CD69 and CD107a staining, respectively. Flow cytometry results indicated that CD69 and CD107a expression of NK cells in the LV-HO1 group were markedly reduced relative to NK cells in the EV1 group (Fig. 4G, Additional file 1: Fig. S5C). On the contrary, knockdown of HO1 in MV4-11 cells upregulated CD69 and CD107a expression of NK cells in the group (Fig. 4H, Additional file 1: Fig. S5D). The addition of CD48 to cells in the LV-HO1 group partly upregulated NK cell CD69 and CD107a expression. Moreover, blocking 2B4 inhibited CD69 and CD107a expression of NK cells in the Si-HO1 group to some extent. Collectively, these data support that overexpression of HO1 in AML cells inhibits NK cell cytotoxicity via targeting the CD48-2B4 axis.

### HO1-overexpression suppressed NK cell cytotoxicity in AML mice

**Fig. 5:**
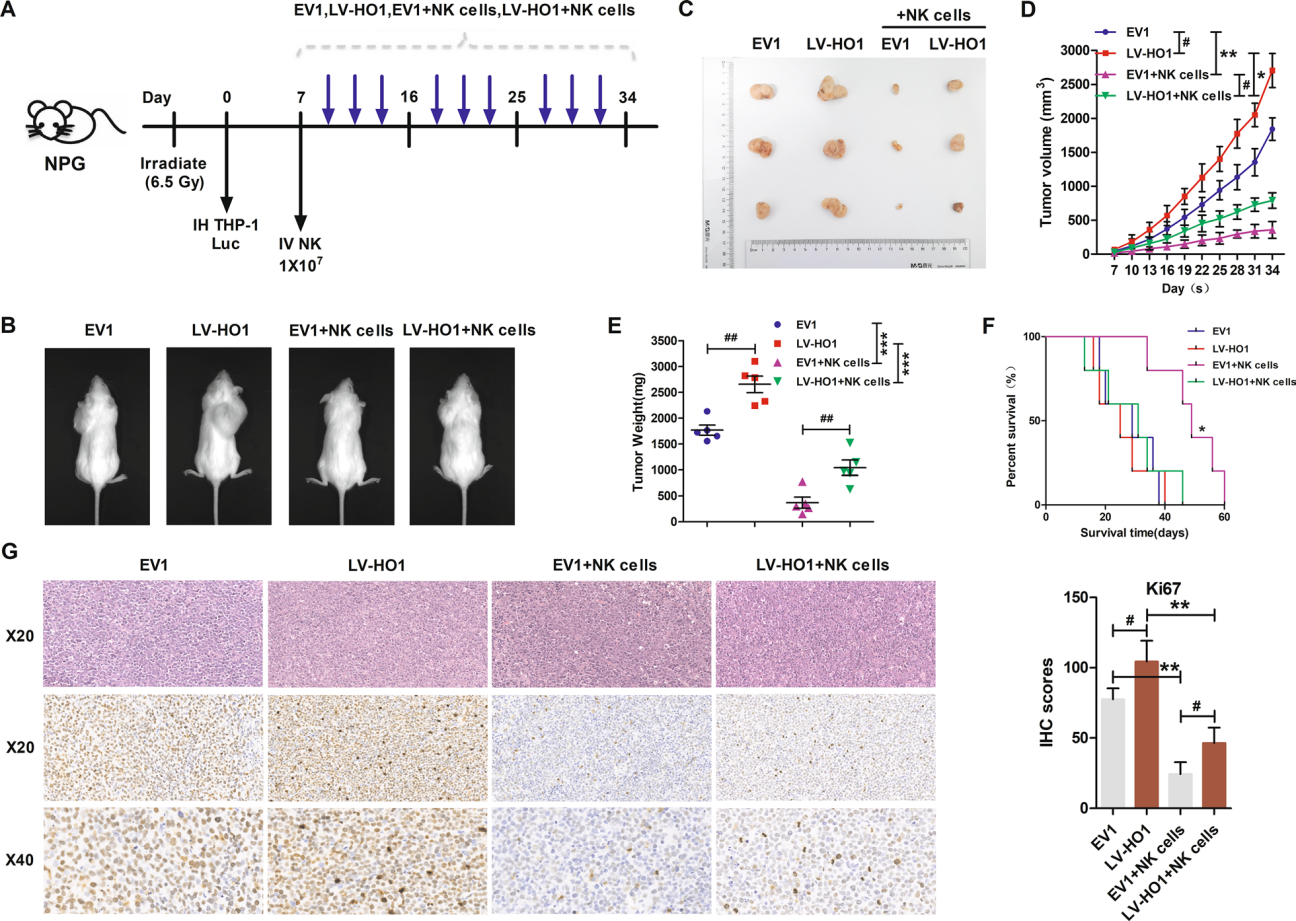
AML cell-derived xenograft (CDX) mice overexpressing HO1 were resistant to the killing effect of NK cells. **A** Schematic illustration showing the experimental design of THP-1luc mice experiments. **B** Representative tumor-bearing mice images in indicated cells. **C** Subcutaneous xenograft images from mice in EV1, LV-HO1, EV1 + NK cells, and LV-HO1 + NK cells groups. **D** Tumor volume growth curves for subcutaneous xenografts. **E** Tumor weight change curves for subcutaneous xenografts. **F** Survival curves for the subcutaneous xenografts. **G** HE and Ki67 staining in xenograft tumors mentioned above (HE, ×20 magnification; Ki67, ×20, ×40 magnification). The Ki67 immunohistochemistry scores were shown in histogram. *p < 0.05, **p < 0.01, ***p < 0.001, ^#^p < 0.05 and ^##^p < 0.01 were calculated using the Student’s t-test

NPG xenograft mice models were developed through subcutaneous administration of HO1 or empty vector-transfected THP-1 cells to explore the effects of HO1 in AML cell immune evasion in vivo. NK cells were administered to the mice via the tail vein immediately after the tumor became palpable (Fig. 5A). The results showed that HO1 overexpression caused an increase in tumor proliferation relative to the EV1 group (Fig. 5B, C). HO1 overexpression effectively promoted an increase in tumor volume (Fig. 5D), tumor weight (Fig. 5E), and cell proliferation (Ki67^+^, Fig. 5G) compared with the EV1 group. Notably, treatment with NK cells caused a significant decrease in tumor burden. Kaplan–Meier analyses of the four groups showed that mice transplanted with EV1 cells and treated with NK cells exhibited longer survival, whereas mice in the other three groups had a similar and shorter survival time (Fig. 5F).

**Fig. 6:**
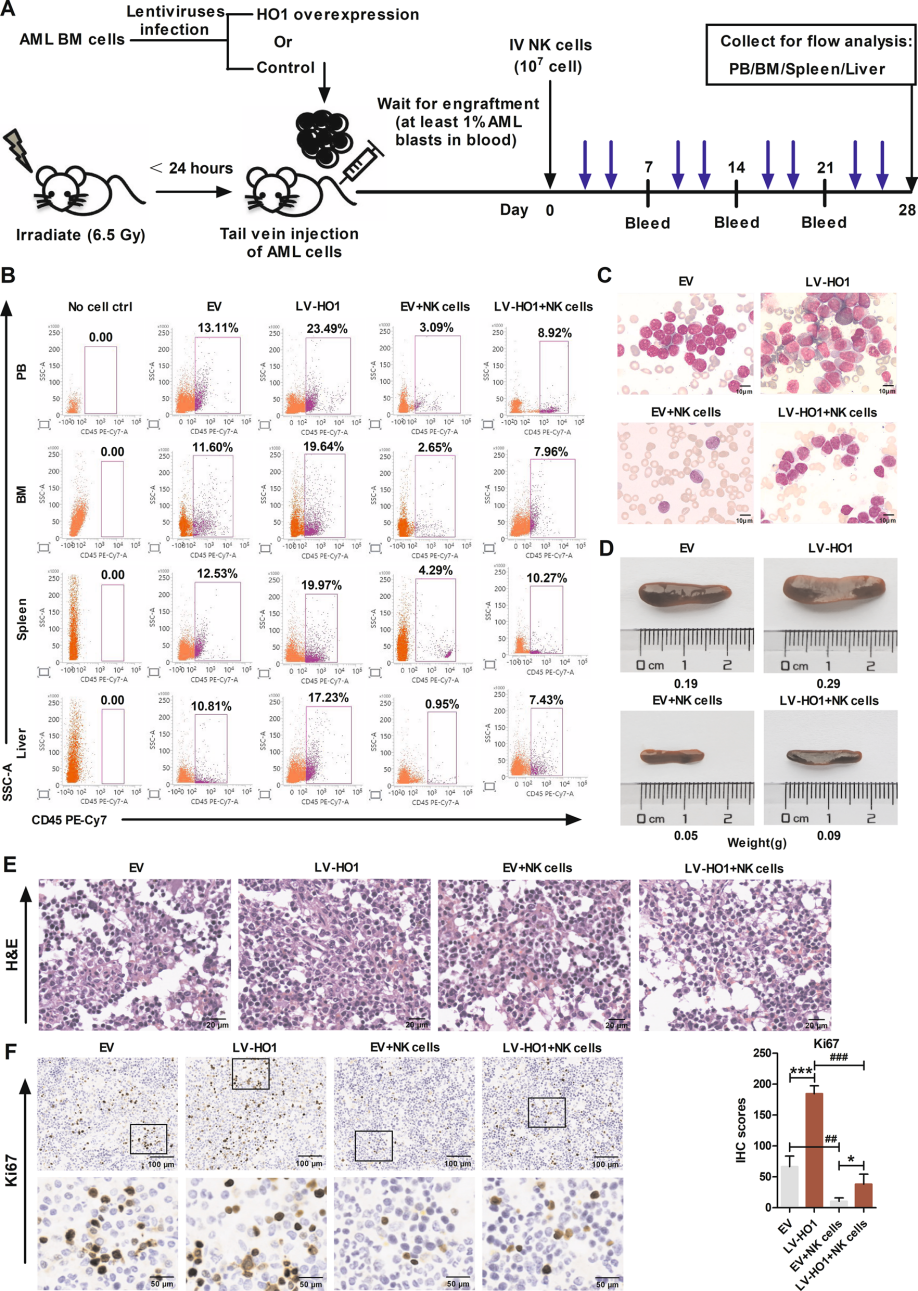
AML PDX mice overexpressing HO1 were resistant to the killing effect of NK cells. **A** Schematic of PDX mouse experiment. **B** Flow cytometry profiles showing human AML blast (CD45+) in PB, BM, spleen, and liver from mice in each group. **C** Wright’s staining analysis under a 100× oil immersion objective for peripheral blood from mice in each group showed AML development (Scale bars = 10 μm). **D** One mouse from every group was sacrificed to obtain images and weights of spleens. **E** Representative HE-stained sections of spleens from all groups. Scale bars: 20 μm. **F** Representative Ki67 stained sections of spleens from all groups. The Ki67 immunohistochemistry scores were shown in histogram. Scale bars: 100 and 50 μm from up to down. *p < 0.05, ***p < 0.001, ^##^p < 0.01 and ^###^p < 0.001 were calculated using the Student’s t-test

Further analysis was conducted using a patient-derived xenograft (PDX) model overexpressing HO1 or control. Bone marrow (BM) primary AML cells were harvested from AML patients and transfected with lentiviruses expressing Luc-tagged control or LV-HO1. Then, these two types of AML primary cells were administered to mice through the tail vein to establish AML mice with overexpression of HO1 or control, respectively. Treatment with NK cells was conducted once each mouse attained ≥ 1% AML blasts in the bloodstream (Fig. 6A). Results showed a higher percentage of AML blasts in peripheral blood (PB), BM, spleen, and livers of the LV-HO1 mice than in mice from the EV group. EV group mice treated with NK cells showed a low abundance of AML blasts in PB, BM, spleen, and liver (Fig. 6B) compared with the other treatment groups. Analysis of bone marrow smear indicated that leukemia cells were significantly decreased after treatment with NK cells (Fig. 6C). At the end of the experiment, the spleens of AML mice were photographed and weighed (Fig. 6D). Larger and heavier spleens observed in the LV-HO1 group indicated that histology of HO1-overexpressed AML mice represented that of advanced cancer stages compared with that of the EV AML mice. Hematoxylin and eosin (HE) staining and immunohistochemistry (IHC) were performed on spleen samples from each group to explore the expression profile of Ki67 (Fig. 6E, F). HO1-overexpressed AML mice showed increased cell proliferation (Ki67+). Moreover, treatment with NK cells significantly decreased cell proliferation. In summary, these findings indicate that HO1 overexpression promoted tumor growth and inhibited the killing effect of NK cells in the AML mice model.

### HO1 downregulated CD48 expression through Sirt1

**Fig. 7:**
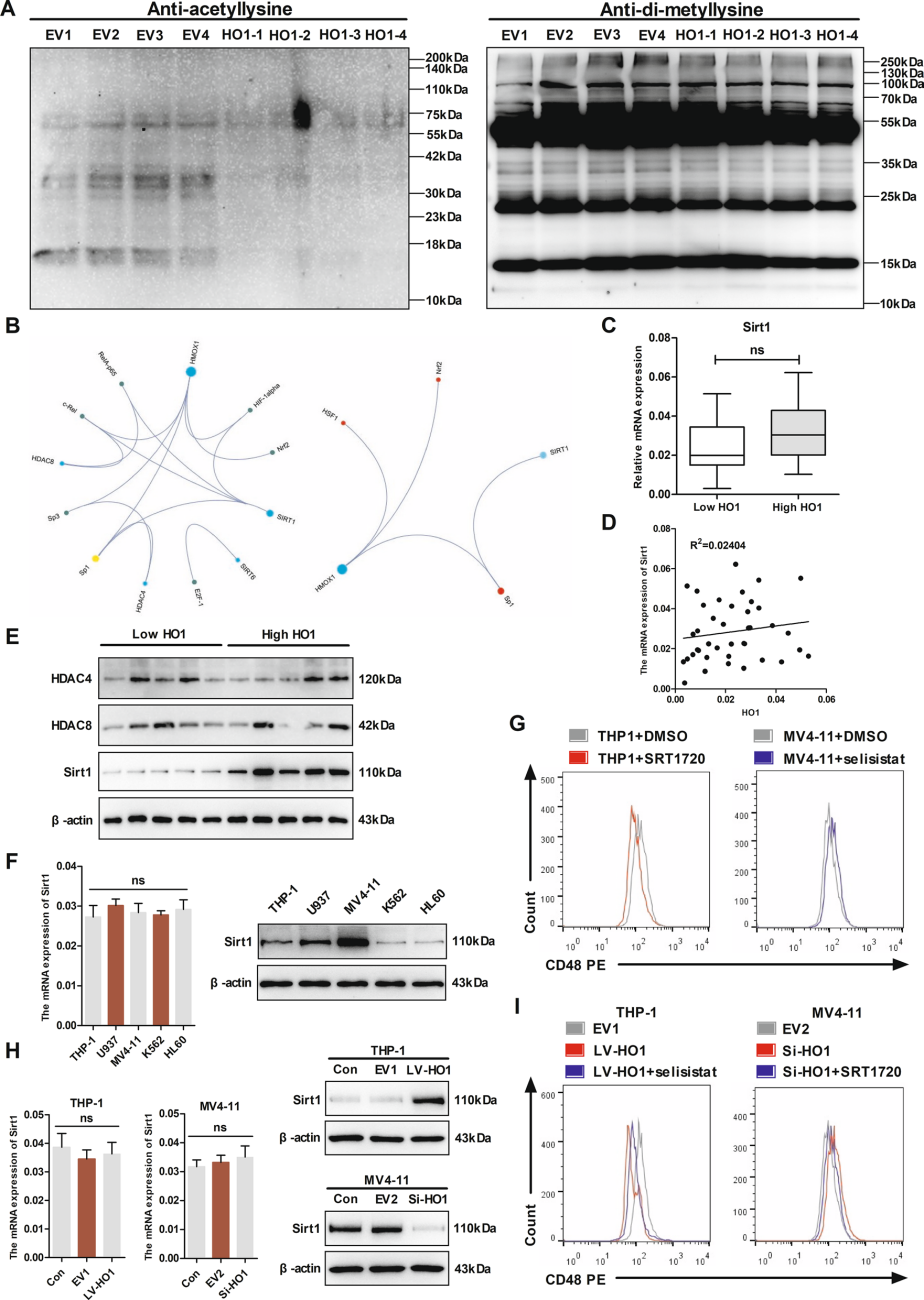
HO1 downregulates CD48 through modulation of Sirt1. **A** Proteins from THP-1 cells transduced with empty vector and HO1 were isolated and analyzed by western blotting using antibodies against acetyllysine or di-methyllysine. **B** Network diagrams for HDACs association studies related to HO1 were generated using GCBI online tool. **C** qRT-PCR assay of Sirt1 gene levels in HO1 high/low expression groups (n = 40). **D** Correlation between HO1 and Sirt1 expression in AML samples as evaluated by qRT-PCR. **E** HDAC4, HDAC8, and Sirt1 protein levels in AML samples as determined by western blotting (n = 10). **F** Sirt1 mRNA and protein expression levels in leukemia cell lines as evaluated by qRT-PCR and western blotting. **G** Left graph: flow cytometry analysis of CD48 expression in THP-1 cells treated with DMSO (gray line) or with SRT1720 (red line). Right graph: flow cytometry analysis of CD48 expression in MV4-11 cells treated with DMSO (gray line) or with selisistat (blue line). **H** The mRNA and protein level of Sirt1 was detected in THP-1 cells overexpressed HO1 and MV4-11 cells silenced HO1. **I** Left graph: flow cytometry evaluation of CD48 levels in THP-1 cells transduced with LV-HO1 (red line) or with an empty vector (gray line). LV-HO1 cells treated with selisistat (blue line) are also shown in this graph. Right graph: flow cytometry evaluation of CD48 levels in MV4-11 cells transduced with Si-HO1 (red line) or an empty vector (gray line). Si-HO1 cells treated with SRT1720 (blue line) are also shown in this graph. Statistical differences were determined using the Student’s t-test. ns, no significance

To investigate the mechanisms by which HO1 downregulates CD48, we reviewed the relevant literature. Elias et al. reported the downregulation of CD48 by oncogenic proteins in AML, facilitated by histone deacetylases (HDACs) [32]. As we know, histone acetylation is an important determinant of gene expression [33]. Considering the research of Elias et al., we hypothesized that HO1 also could downregulate CD48 expression by recruiting HDACs. Increased enzymatic activity of HDACs can result in decreased histone acetylation, while deacetylated histones are often associated with gene repression [34]. Therefore, we first assessed the acetylation levels after overexpressing HO1 in AML cells for preliminary validation. THP-1 cells were transduced with lentivirus encoding vector control and LV-HO1. Proteins were extracted, and pan-acetylation was analyzed using the antibody against acetyllysine. A low pan-acetylation level was observed in THP-1 cells overexpressing HO1 (Fig. 7A, left graph). On the contrary, overexpression of HO1 exhibited a minimal effect on pan-methylation of proteins in THP-1 cells (Fig. 7A, right graph). Since HDACs are enzymes that deacetylate histones, it was important to determine if HO1 affected HDACs. Next, a literature review was conducted on studies that assessed the relationship between HDACs and HO1 by searching PubMed and Gene-Cloud Biotechnology Information (GCBI) databases. The results showed that HO1 could interact with transcription factors such as c-Rel, Sp3, Sp1, or HIF-1α, thus affecting the expression of HDACs, such as HDAC4, HDAC8, or Sirt1 (Fig. 7B).

Then, we performed a series of experiments in AML patients to initially validate the above findings. The results demonstrated that Sirt1 was not correlated with HO1 in mRNA levels (Fig. 7C, D). While Sirt1 was increased in HO1-high expressing AML patients in protein levels (Fig. 7E, Additional file 1: Fig. S6A). Notably, HDAC4/HDAC8 was not correlated with HO1 in mRNA/protein levels (Fig. 7E, Additional file 1: Fig. S6A–C). As a histone deacetylase, histone deacetylation induced by Sirt1 leads to gene silencing [34]. Therefore, we hypothesized that HO1 could downregulate CD48 expression by recruiting Sirt1.

Further analysis was conducted to explore whether treatment with Sirt1 inhibitor (selisistat) or activator (SRT1720) affects the expression of CD48. Sirt1 expression in leukemia cell lines was first determined. Our findings showed that Sirt1 protein levels were elevated in MV4-11 cells relative to other cell lines, while the expression of Sirt1 in THP-1 was the lowest in standard AML cell lines (Fig. 7F, Additional file 1: Fig. S6D). SRT1720 significantly downregulated the expression of CD48 in THP-1 (Fig. 7G, left graph), whereas selisistat upregulated CD48 expression in MV4-11 (Fig. 7G, right graph).

Sirt1 and CD48 expression profiles were explored in THP1 and MV4-11 transfected cells to assess whether HO1 downregulated CD48 by recruiting Sirt1. HO1 overexpression significantly increased Sirt1 protein levels and downregulated CD48 expression. Notably, the addition of selisistat rescued CD48 expression in cells expressing LV-HO1 to some extent by weakening the negative regulation of CD48 by HO1. Sirt1 protein level was downregulated, whereas CD48 expression was upregulated by the downregulation of HO1 in MV4-11 cells. The addition of SRT1720 decreased CD48 expression in Si-HO1 by restoring the negative regulation of CD48 by HO1 (Fig. 7H, I, Additional file 1: Fig. S6E). These data support that HO1 downregulates CD48 expression through recruitment of Sirt1, and selective inhibitors of Sirt1 can reverse this effect.

### H3K27ac mediated Sirt1 and HO1 action regarding CD48 expression

**Fig. 8:**
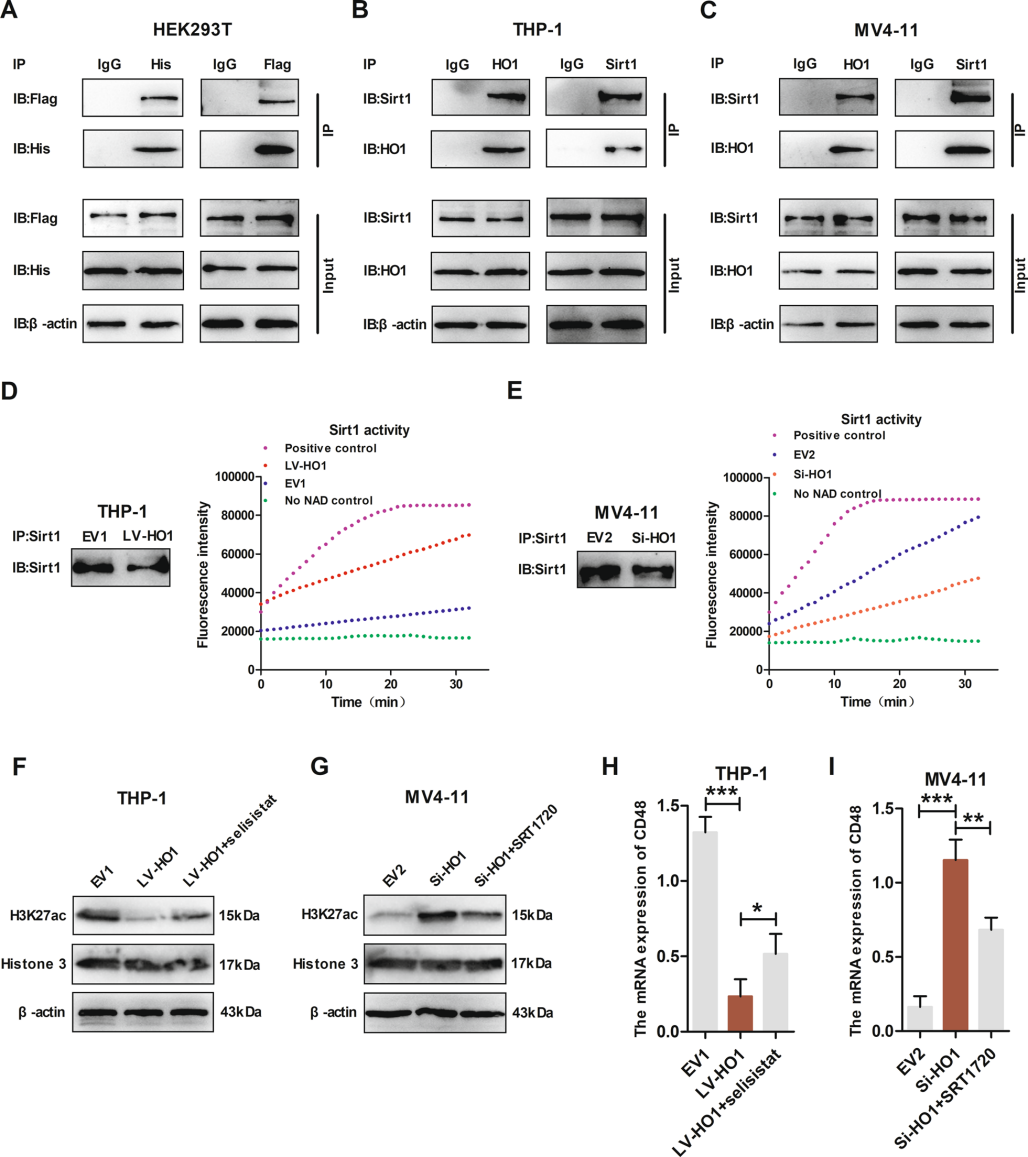
H3K27ac mediated Sirt1 and HO1 action regarding CD48 expression. **A** HEK293T cells were transfected with the His-tagged HO1 and Flag-tagged Sirt1 plasmids. Cell lysates were immunoprecipitated with anti-His-HO1 or anti-Flag-Sirt1 antibody and immunoblotted with anti-Flag-Sirt1 or anti-His-HO1 antibody, respectively. **B** THP-1 cell lysates were immunoprecipitated with anti-HO1 or anti-Sirt1 antibody and immunoblotted with anti-Sirt1 or anti-HO1 antibody, respectively. **C** MV4-11 cell lysates were immunoprecipitated with anti-HO1 or anti-Sirt1 antibody and immunoblotted with anti-Sirt1 or anti-HO1 antibody, respectively. **D** Sirt1 deacetylation activity assay of Sirt1 immunoprecipitated from extracts of THP-1 expressing EV1 or LV-HO1. Proteins loaded were analyzed by western blotting. This experiment has been repeated for three times. **E** Sirt1 deacetylation activity assay of Sirt1 immunoprecipitated from extracts of MV4-11 expressing EV2 or Si-HO1. Proteins loaded were analyzed by western blotting. This experiment has been repeated for three times. **F** H3K27ac levels in EV1 and LV-HO1 THP-1 cells were quantified by western blotting analysis. LV-HO1 cells treated with selisistat are also shown in this graph. **G** H3K27ac levels in EV2 and Si-HO1 MV4-11 cells were quantified by western blotting analysis. Si-HO1 cells treated with SRT1720 are also shown in this graph. **H** CD48 mRNA levels in EV1 and LV-HO1 THP-1 cells were quantified by qRT-PCR analysis. LV-HO1 cells treated with selisistat are also shown in this graph. **I** CD48 mRNA levels in EV2 and Si-HO1 MV4-11 cells were quantified by qRT-PCR analysis. Si-HO1 cells treated with SRT1720 are also shown in this graph. Statistical differences were determined using the Student’s t-test. *p < 0.05, **p < 0.01, ***p < 0.001

As shown in Fig. 7H, overexpression of HO1 increased Sirt1 protein levels. To investigate how HO1 interacts with Sirt1, HEK293T cells were transfected with plasmids expressing His-tagged HO1 and Flag-tagged Sirt1. Immunoprecipitation assays of His-tagged HO1 and Flag-tagged Sirt1 revealed co-precipitation of HO1 with exogenous Sirt1 (Fig. 8A). In addition, co-immunoprecipitation assays demonstrated that HO1 could form a complex with endogenous Sirt1 in THP-1 and MV4-11 cells (Fig. 8B, C). Taken together, these data suggest that HO1 binds Sirt1 in AML cells. To further investigate the mechanisms of HO1 in regulating Sirt1 expression after their binding, we assessed the deacetylation activity of Sirt1 immunoprecipitated from extracts of transduced AML cells. The results demonstrated that knockdown of HO1 significantly decreased the activity of Sirt1 in MV4-11, while overexpression of HO1 markedly increased the activity of Sirt1 in THP-1 (Fig. 8D, E). Collectively, these lines of evidence suggest that in AML cells, HO1 directly interacted with Sirt1 and increased its expression and deacetylase activity.

We reviewed the relevant literature to identify which histone lysine residues are regulated by Sirt1 in AML cells. Umemoto et al. reported that increased histone 3 lysine 27 acetylation (H3K27ac) levels in hematopoietic stem cells enabled the promotion of CD48 transcription and expression [35]. To elucidate whether Sirt1 inhibits CD48 expression by repressing H3K27ac, HO1 transduced AML cells were examined. The results revealed that HO1 overexpression markedly decreased the H3K27ac and CD48 mRNA levels. Correspondingly, the addition of selisistat rescued H3K27ac and CD48 mRNA levels in cells expressing LV-HO1 to a certain extent (Fig. 8F, H, Additional file 1: Fig. S7A), while H3K27ac and CD48 mRNA levels were upregulated by the downregulation of HO1 in MV4-11 cells. Moreover, SRT1720 decreased H3K27ac and CD48 mRNA levels in Si-HO1 to some extent (Fig. 8G, I, Additional file 1: Fig. S7B). Accordingly, H3K27ac is key for regulating CD48 expression by both HO1 and Sirt1. These results were consistent with our hypothesis that overexpression of HO1 increased Sirt1 in AML cells enabling histone H3K27 deacetylation to suppress CD48 transcription and expression (Additional file 1: Fig. S8).

## Discussion

This study uncovered the biological and immunological roles of HO1 in AML. HO1 is widely acknowledged to be highly expressed in AML patients and negatively correlated with CD48 levels. The present study’s findings indicated that HO1 specifically downregulates CD48 levels, a ligand of the natural killer (NK) cell-activating receptor 2B4, thus decreasing the cytotoxic effect of NK cells. Mechanistic investigations found that HO1 directly interacted with Sirt1 and increased its expression and the deacetylase activity. Overexpression of HO1 increased Sirt1 in AML cells enabling histone H3K27 deacetylation to suppress CD48 transcription and expression. Administration of Sirt1 inhibitor could restore the expression of CD48. The prognosis of AML patients with elevated HO1 levels is generally poor, and most of them die as a result of refractory (initial chemotherapeutic resistance) or relapsed AML. Although the poor prognosis may be associated with other non-immune mechanisms, these findings suggest that targeting the HO1/Sirt1/CD48-2B4 axis is a promising strategy for successfully treating AML.

As described before [18], HO1 is an enzyme that breaks down dying cells to release heme and consequently produces biliverdin, Fe2+ and CO. The CO is closely related to the p38 MAPK, STAT1/3, and NFκB signaling pathways, proposed for HO1-mediated cytoprotective and anti-inflammatory effects. However, little is currently known about how HO1 directly interacts with Sirt1 and increases its expression and the deacetylase activity. Indeed, further molecular mechanisms of HO1 in regulating the expression and deacetylase activity of Sirt1 are warranted in the future.

Interestingly, this study revealed an epigenetic modification mechanism in AML cells that histone deacetylation by Sirt1 is essential for HO1-induced immune evasion (Additional file 1: Fig. S8). Indeed, it is well-established that histone deacetylation downregulates gene expression by causing chromatin folding and decreasing transcription factor access [36]. Therefore, how transcription factors recruit Sirt1 to the CD48 promoter remains unclear. In future studies, DNA pulldown coupled with mass spectrometry (MS) analysis should be performed to search for putative transcription factor-binding sites.

There is ample evidence suggesting that HO1 mediates M1/M2 macrophage polarization [17–20]. The present study further deepened our current understanding of the immunological roles of HO1 by demonstrating NK dysfunction caused by HO1. Innate immunity mediated by NK cells is reportedly critical for attacking tumors, especially when acquired immunity is exhausted and dysfunctional [37, 38], and tumor cells express no or rare MHC necessary for the induction and activation of T cells [39]. Therefore, anti-HO1 therapy that restores NK immunity may be widely useful for treating cancer, especially in AML patients with high HO1 and Sirt1 expression.

## Conclusions

In the present study, we elucidated biological and immunological mechanisms underlying refractory AML using clinical specimens, human AML cell lines, and mouse AML models. Collectively, HO1 promotes NK cell dysfunction in AML. Therefore, restoring NK cell function by inhibiting HO1 activity is a potential immunotherapeutic approach against AML.

## Supplementary Information

**Additional file 1.** **Figure S1**. Correlation between HO1 and immune cells. **Figure S2**. Correlation between HO1 and immune checkpoint/ligand molecules. **Figure S3**. Correlation between HO1 and immune checkpoint/ligand molecules. **Figure S4**. Immune cell distributions (CD4+ T, CD8+ T, B, and NK cells) in purified primary NK cells. **Figure S5**. Overexpression of HO1 in AML cells inhibited NK cell cytotoxicity via targeting the CD48-2B4 axis. **Figure S6**. Correlation between HO1 and HDACs expression. **Figure S7**. H3K27ac mediated the effects of HO1 and Sirt1 on CD48 expression. **Figure S8**. Schematic representation of HO1 mediated immune evasion to NK cells in AML. **Table S1**. Characteristics of patient samples. **Table S2**. Antibodies used for flow cytometry. **Table S3**. The characteristics of the primers used for qRT-PCR.

## Data Availability

The data used and/or analysis during the current study are available from the corresponding author on reasonable request.

## Abbreviations

AML: Acute myeloid leukemia
HO1: Heme oxygenase 1
NK: Natural killer
Sirt1: Sirtuin1
H3K27ac: Histone 3 lysine 27 acetylation
IC: Immune checkpoint
CO: Carbon monoxide
ESTIMATE: Estimation of STromal and Immune cells in Malignant Tumor tissues with Expression data
RNA-seq: Ribonucleic acid sequencing
TCGA: The Cancer Genome Atlas
TIMER: Tumor Immune Estimation Resource
qRT-PCR: Real-Time Quantitative Reverse Transcription PCR
BMNCs: Bone marrow mononuclear cells
mRNA: Messenger RNA
CDX: Cell-derived xenograft
PDX: Patient-derived xenograft
PB: Peripheral blood
IHC: Immunohistochemistry
HDACs: Histone deacetylases
GCBI: Gene-Cloud Biotechnology Information
MS: Mass spectrometry
MHC: Major histocompatibility complex
DMSO: Dimethyl sulfoxide
