# Supplementary material for: Heme oxygenase 1 overexpression induces immune evasion of acute myeloid leukemia against natural killer cells by inhibiting CD48
Source: J Transl Med. 2022 Sep 4;20:394. doi: 10.1186/s12967-022-03589-z (PMC9441067; doi:10.1186/s12967-022-03589-z)
Supplement: Supplementary file 1 — Additional file 1. Figure S1. Correlation between HO1 and immune cells. Figure S2. Correlation between HO1 and immune checkpoint/ligand molecules. Figure S3. Correlation between HO1 and immune checkpoint/ligand molecules. Figure S4. Immune cell distributions (CD4+ T, CD8+ T, B, and NK cells) in purified primary NK cells. Figure S5. Overexpression of HO1 in AML cells inhibited NK cell cytotoxicity via targeting the CD48-2B4 axis. Figure S6. Correlation between HO1 and HDACs expression. Figure S7. H3K27ac mediated the effects of HO1 and Sirt1 on CD48 expression. Figure S8. Schematic representation of HO1 mediated immune evasion to NK cells in AML. Table S1. Characteristics of patient samples. Table S2. Antibodies used for flow cytometry. Table S3. The characteristics of the primers used for qRT-PCR. [file 12967_2022_3589_MOESM1_ESM.docx]

**Additional materials and methods**

**Clinical samples and cell lines**

40 bone marrow specimens were collected through a simple random sampling method from AML patients attending the Affiliated Hospital of Guizhou Medical University from September 2020 to May 2022. Normal samples were donated by healthy individuals. Patient clinical data are shown in Table S1. Patient samples at diagnosis and relapse were collected prior to treatment. The Institutional Research Ethics Committee approved this study, and informed consent was acquired before the collection of samples. Primary patient samples were purified with Ficoll-Hypaque density centrifugation and then cultured using the StemSpan Leukemic Cell Culture Kit (STEMCELL Technologies).

Human cell lines, including THP-1, U937, MV4-11, K562, HL60, and HEK293T, were purchased from Leibniz Institute DSMZ-German Collection of Micro-organisms and Cell Cultures (DSMZ). The cell lines were assessed for contamination with mycoplasma and were confirmed by short tandem repeat profiling. Then, they were cultured in 10% fetal bovine serum (FBS)-supplemented RPMI-1640 medium with 100 units/mL penicillin and 100 mg/mL streptomycin. Incubation was done at 37 °C in a 5% CO_2_ humid environment.

**Reagents and antibodies**

BD FACS Lysing Solution was purchased from BD Biosciences. Antibodies for flow cytometry staining are presented in Table S2. The RPMI 1640 medium and FBS were acquired from Gibco (Carlsbad, CA, USA). Recombinant human CD48 protein was purchased from T&L Biological Technology. Blocking anti-2B4 antibodies were provided by R&D systems. The selective Sirt1 inhibitor selisistat and activator SRT1720 were purchased from Selleck Chemicals (USA). Western blot was conducted using anti-HO1, anti-Sirt1, anti-H3K27ac, and Histone 3 antibodies purchased from Abcam. Anti-HDAC4, anti-HDAC8, as well as anti-β-actin antibodies were purchased from Huabio (Hangzhou, China). Anti-acetyllysine and anti-di-methyllysine antibodies were purchased from PTM BIO (Hangzhou, China). Anti-His, anti-Flag antibodies, and protein A/G PLUS-agarose were purchased from Santa Cruz Biotechnology.

**Tumor immune infiltration analysis**

The abundance of immune infiltrates was estimated using a web server for comprehensive analysis of tumor infiltrating immune cells, named the Tumor Immune Estimation Resource (TIMER) database [1].

**ESTIMATE analysis**

Immune and stromal scores were calculated using the ESTIMATE (Estimation of STromal and Immune cells in Malignant Tumor tissues with Expression data) algorithm. The ESTIMATE is an algorithm that provides scores for the level of stromal cells present and the infiltration level of immune cells in tumor tissues by calculating specific molecular biomarker expression in immune and stromal cells to predict the tumor microenvironment. The stromal score captures the presence of stroma, the immune score represents the infiltration of immune cells, and the ESTIMATE score infers tumor purity and is equal in number to stromal score and immune score [2].

**The Cancer Genome Atlas (TCGA) database**

For pan-cancer data of TCGA and Genotype-Tissue Expression (GTEx) databases, differential HO1 mRNA expressions between various tumors and normal tissues were analyzed using T-test and their visualization was carried out by R software package “ggplot2”. The correlation heatmaps between HO1 expression and immune checkpoint/ligand genes were plotted using R software package “corrplot”.

**T-cell, B-cell and NK cell (TBNK) assay**

The Agilent QuadriTest 4-color Immunophenotyping Kit and a BD FACSLyric flow cytometer were used to determine human lymphocyte percentage in whole blood. Identification of T lymphocytes was done by detecting CD3+ cells, T cytotoxic cells were identified as CD3+ CD8+, T helper cells were identified as CD3+ CD4+, B cells were detected as CD3− CD19+, and NK cells were identified as CD3− CD16+ CD56+.

**Flow cytometry staining**

Qualitative and quantitative levels of various markers were explored by multiparametric flow cytometry. Staining of samples was done using various antibody combinations against HO1, CD117, CD33, CD45, 2B4, CD56, CD48, HAVCR2 (CD366), CD86, CD200R1 (CD200R), TNFRSF8 (CD30), CD40, TNFRSF9 (CD137), PDCD1LG2 (CD273), CTLA4 and VSIR (VISTA). The Fluorescence minus one, unstimulated, and autofluorescence controls were included for accurate placement of the gate. Clinical specimens from the same patient but obtained at various time points were assessed on the same day to reduce variations. Blocking buffer A [50% fluorescence-activated cell sorting buffer (FACS) + 50% human serum] was used to block Fc receptors before surface marker staining. Cells were stained with appropriate surface antibodies, then fixed and permeabilized for 20 min using Cytofix/Cytoperm reagent (BD Biosciences), and subsequently stained with antibodies to intracellular molecules HO1. All acquisitions were performed on a FACSLyric device (BD Biosciences), while the data were analyzed by BD FACSuite v1.3 software (BD Biosciences) and Flowjo software (TreeStar).

**Quantitative real-time PCR (qRT-PCR)**

Extraction of total RNAs from cells was done using the Trizol reagent (Invitrogen, Carlsbad, CA, USA), according to the manufacturer's instructions. Real-time PCR was conducted using the SYBR Green PCR Master Mix (TianGen Biotech, Beijing, China) on the PRISM 7500 real-time PCR detection system (ABI, USA). The qRT-PCR primers (Sangon Biotech, Shanghai, China) are presented in Table S3.

**Western blot analysis**

This assay was conducted as previously described [3].

**Lentiviral transduction**

The Lentivirus-hU6-MCS-Ubiquitin-firefly-Luciferase-IRES-puromycin was acquired from Genechem Co., Ltd. (Shanghai, China). The human HO1 overexpression clone lentiviral particle (LV-HO1) and human HO1-RNAi were transduced into AML cells according to the manufacturer's protocol. The controls were cells (MV4-11 and THP-1) transfected with the empty vector. Stable THP-1 and MV4-11 cell lines expressing LV-HO1 or Si-HO1 were selected by puromycin (1.5 and 2 μg/ml, respectively) after expansion and maintenance for 5 days in RPMI-1640 medium with 10% FBS.

**Human NK cell extraction and cultures**

Enriched NK cells were isolated from healthy donor peripheral blood mononuclear cells (PBMCs) using the NK cell culture kit (T&L Biological Technology, Cat. No: AS-01). Populations with over 80% CD3− CD16+ CD56+ NK cells were chosen for subsequent assays (Fig. S4). All experiments were performed based on the principles of the Declaration of Helsinki. Informed consent (written) was acquired from all donors before blood collection, while ethical approval was granted by the Guizhou Medical University.

**Leukemia cells and NK cell co-culture assay**

As effector cells, purified NK cells were co-cultured in the presence of Luciferase-tagged THP-1 or MV4-11 cells (target cells) at varying effector/target cell ratios (1:1, 2:1, 4:1, 10:1). All culture experiments were performed in triplicate wells for 2 h at 37°C. Survival outcomes for transfected cells were evaluated by luminescence after adding luciferin (50 ng; Beyotime, Beijing, China). Cell survival outcomes for all groups were normalized to the survival levels of untreated cells.

**NK cell cytotoxic assays**

The NK cell-associated cytotoxicity was explored by the Cytotoxicity Detection Kit PLUS (Roche, Reinach, Switzerland) and was based on the determination of LDH from damaged cells. Purified NK cells (as effector cells) were incubated with transduced THP-1 or MV4-11 cells (as target cells) at various effector cell/target cell ratios (1:1, 2:1, 4:1, 10:1) in 96-well plates for 3.5 h. Test samples were prepared in triplicates. Reaction mixtures, as well as the stop solution, were sequentially added to each well. Sample absorbance was determined at 490 nm using an ELISA reader. The levels (%) of NK cell-associated cytotoxicity were determined based on the ODs as Cytotoxicity (%) = (effector/target cell mix－effector cell control－low control)/(high control－low control) × 100.

**Activation of NK cells against tumor cell lines**

NK cell activation was explored by flow cytometry using CD107a and CD69 as degranulation and activation markers, respectively. NK cells at a cell density of 5×10^4^ were co-cultured with 5×10^4^ transduced THP-1 or MV4-11 cells in the presence of CD107a-PE-Cy7 (Biolegend) or CD69-PE (BD Pharmingen) antibodies at 37°C under 5% CO_2_ for 6 h in a 96-U-bottom plate. 1µl of 100× monensin (Biolegend) and Brefeldin A (Biolegend) were supplemented into every well after the first hour of incubation. NK cells alone group was set as the negative control. After incubation, the cells were washed twice using staining a buffer and then stained using a mixture of anti-CD45-PerCP (BD Pharmingen) and anti-CD56-APC (Beckman Coulter) antibodies at 4°C for 30 min. Then, they were washed twice and evaluated using a FACSLyric flow cytometer.

**In vivo models**

The Guizhou Medical University Laboratory Animal Center permitted the use of animals in this study. Male 6-8-week-old NOD/SCID IL‐2Rγ null (NPG) mice were obtained from the Beijing Vital River Laboratory Animal Technology. The serial number for the production license for the laboratory animals (SCXK) was SCXK-2019-0002.

Stably transfected THP-1luc cells were resuspended in PBS (5×10^6^ cells/100 μL) and subcutaneously administered to the mice randomized into 4 groups: EV1, LV-HO1, EV1+NK cells, and LV-HO1+NK cells. Mice were treated with primary NK cells (1×10^7^/mouse) once the tumors were visible or palpable, through injections via the tail vein every three days for a total of 9 treatments. Mice were placed on the BLT In-Vivo Imaging System (BLT Photon Tech., Guangzhou, China) platform for observation. Tumors diameter and weights were determined every three days.

Primary cells were isolated from AML patient bone marrow (BM) to establish a patient-derived xenograft (PDX) model. Proliferation and induction of primary AML cells were conducted in vitro. Furthermore, Luc-tagged lentiviral infection was performed to obtain two subtypes of AML-BM primary cells, HO1 overexpression and control groups. Irradiated (6.5Gy) 6-8-week-old NPG mice were intravenously administered with two types of primary AML cells (10 per group, 2×10^6^ cells transplanted in each mouse). Progression of engrafted AML was assessed through weekly bleeds to evaluate the abundance of CD45+/CD33+ blast cells until at least 1% human AML was observed in the blood (in approximately 6 weeks). The relevant groups received 10^7^ human NK cells twice a week for 4 weeks by tail vein injection from Day 0 to mimic clinical practice. Human CD45+ cells (leukemia load) in the peripheral blood (PB), spleen, BM, and liver were assessed by flow cytometry and IHC analyses on Day 28.

**Immunohistochemistry (IHC) staining**

IHC staining using antibodies against Ki67 was conducted to determine protein levels. Quantification of protein expression level was conducted using ImageJ software.

**Wright staining**

Human primary leukemia cells were stained with Wright staining solution and were observed under a microscope (Leica, Germany).

**Data source**

Networks for HDACs association studies related to HO1 were generated by retrieving studies on human genetic associations in Gene-Cloud Biotechnology Information (GCBI) and PubMed databases.

**Immunoprecipitation assay**

Immunoprecipitation assays were performed to check the possible interaction between HO1 and Sirt1. Both HEK293T cells transfected with His-tagged HO1 and Flag-tagged Sirt1 plasmids and THP-1/MV4-11 cells were lysed with RIPA and a protease inhibitor mixture for 30 minutes. The cell lysates were centrifuged at 12,000 rpm for 10 min at 4 °C and then (400 μg) incubated with 2 μg of indicated primary antibody at 4 °C overnight on a rotating wheel. Twenty microliters of resuspended protein A/G PLUS-agarose were added at 4 °C for 1 h on the rotating wheel. The beads were collected by centrifugation at 12000g for 1 min at 4 °C and washed twice with RIPA. After a final wash, the beads were resuspended in 40 μL of 1× loading buﬀer. Samples were boiled for 4-5 min and subjected to western blot analysis.

**Sirt1 deacetylation activity assay**

Cells were lysed in a buffer containing 20 mM Tris pH 7.5, 137 mM NaCl, 1 mM MgCl_2_, 1 mM CaCl_2_, 1% NP-40 and 10% glycerol supplemented with 1:100 Halt protease and phosphatase inhibitor cocktail (Thermo Scientific) at 4 °C for 1 h. The supernatant was incubated overnight with antibody-conjugated agarose beads at 4 °C. The next day, the beads were washed four times for 10 min at 4 °C, 5 μl buffer was added, and the beads were subjected to Sirt1 activity detection using a Sirt1 activity assay kit (Abcam, ab156065). The deacetylation activity of the samples was determined from the average slopes of the fluorescence intensity curves, which were calculated through lineage regression analyses.

**Statistical analysis**

Data analyses were conducted by GraphPad Prism 8.0 software (GraphPad Software, Inc, USA). The normal distribution of clinical data was determined by the Shapiro-Wilk normality test. Data are shown as mean ± SD for n = 3. An unpaired two-tailed Student's t-test was used to assess between-group differences. Survival data were presented as a Kaplan-Meier survival plot. A p-value ≤0.05 was statistically significant.

**Additional figures**


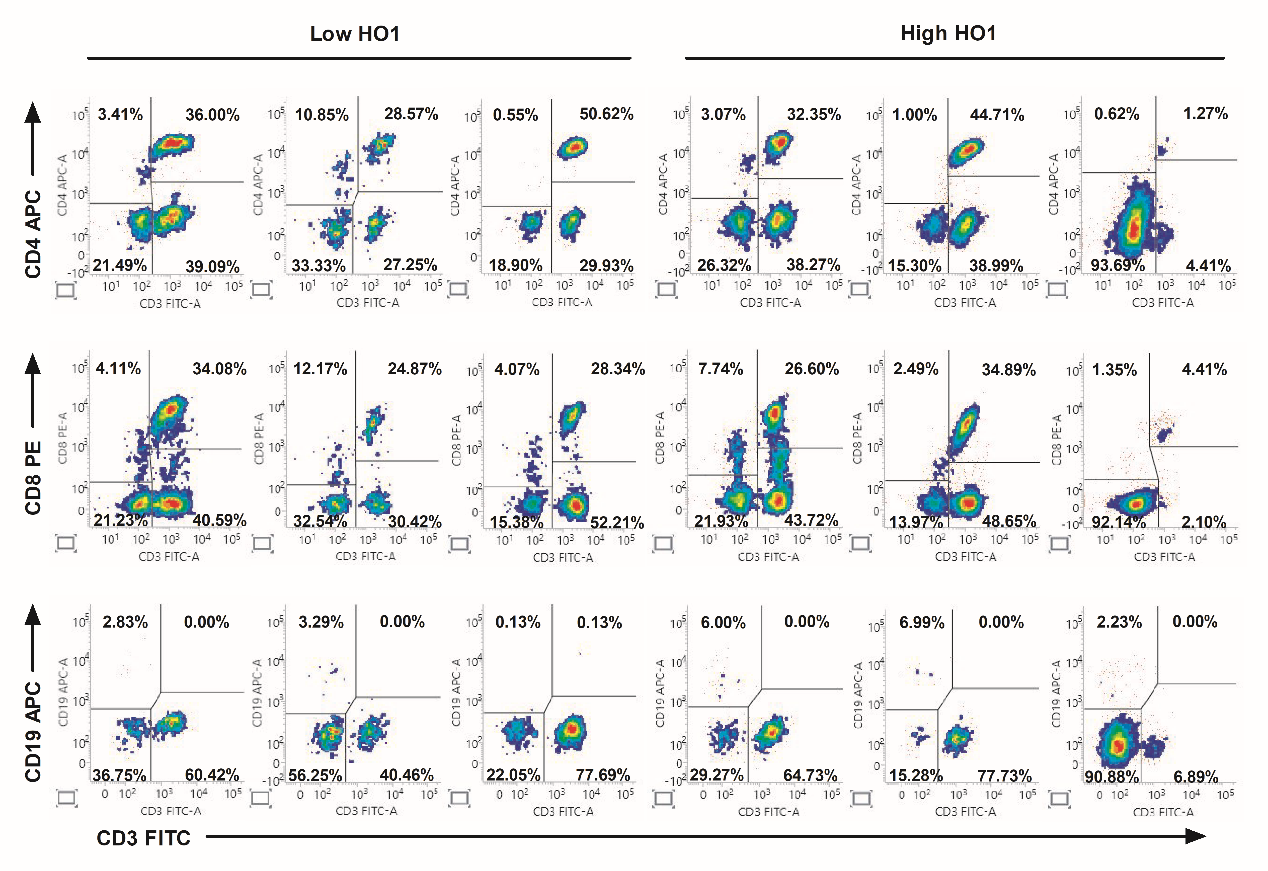


**Fig S1. Correlation between HO1 and immune cells.**

Percentage of CD4+ T cells (CD3+ CD4+), CD8+ T cells (CD3+ CD8+) and B cells (CD3− CD19+) in HO1 high/low AML specimens as determined by flow cytometry.


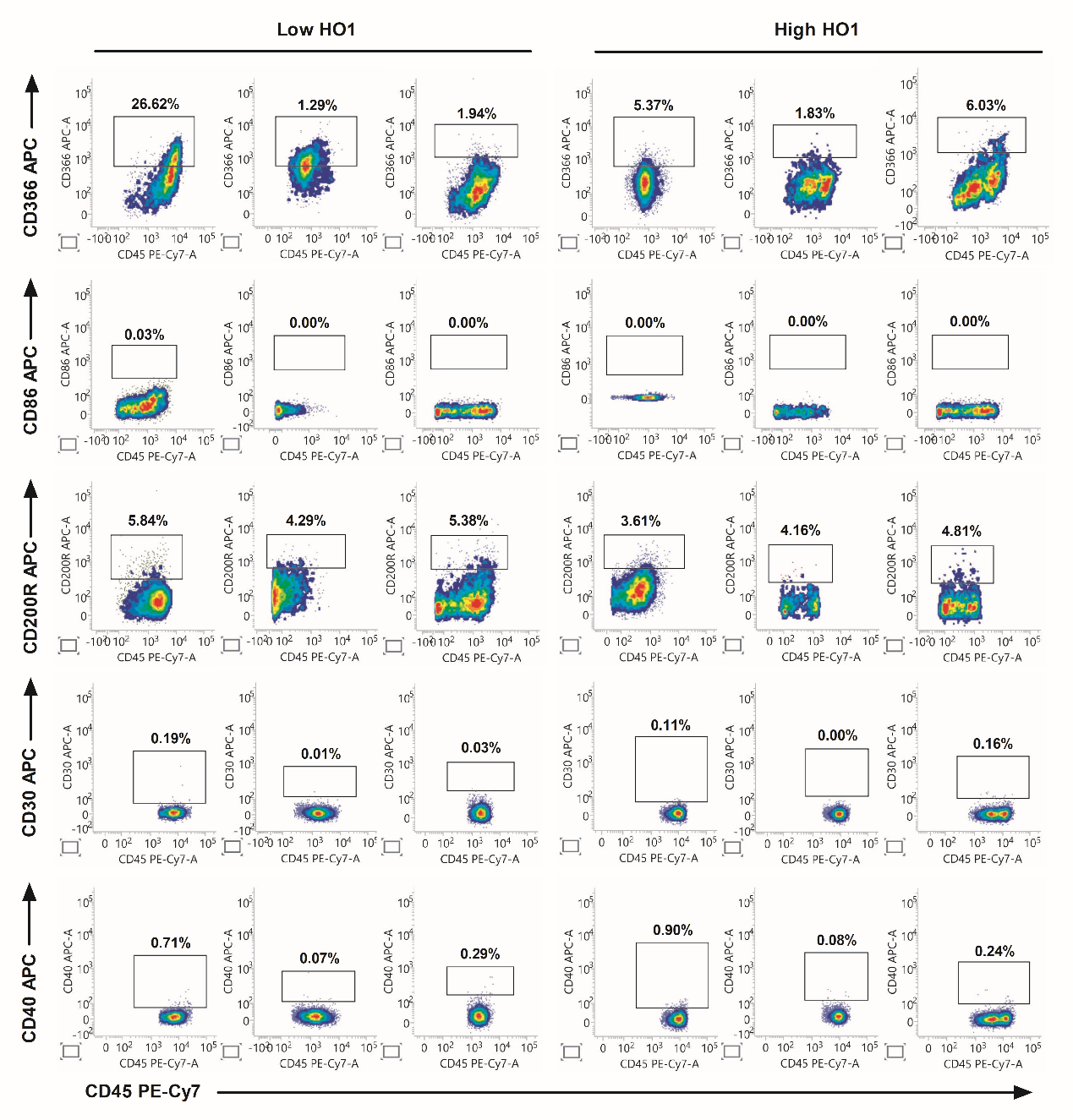


**Fig S2. Correlation between HO1 and immune checkpoint/ligand molecules.**

CD366, CD86, CD200R, CD30 and CD40 expression levels in HO1 high/low AML specimens as determined by flow cytometry.


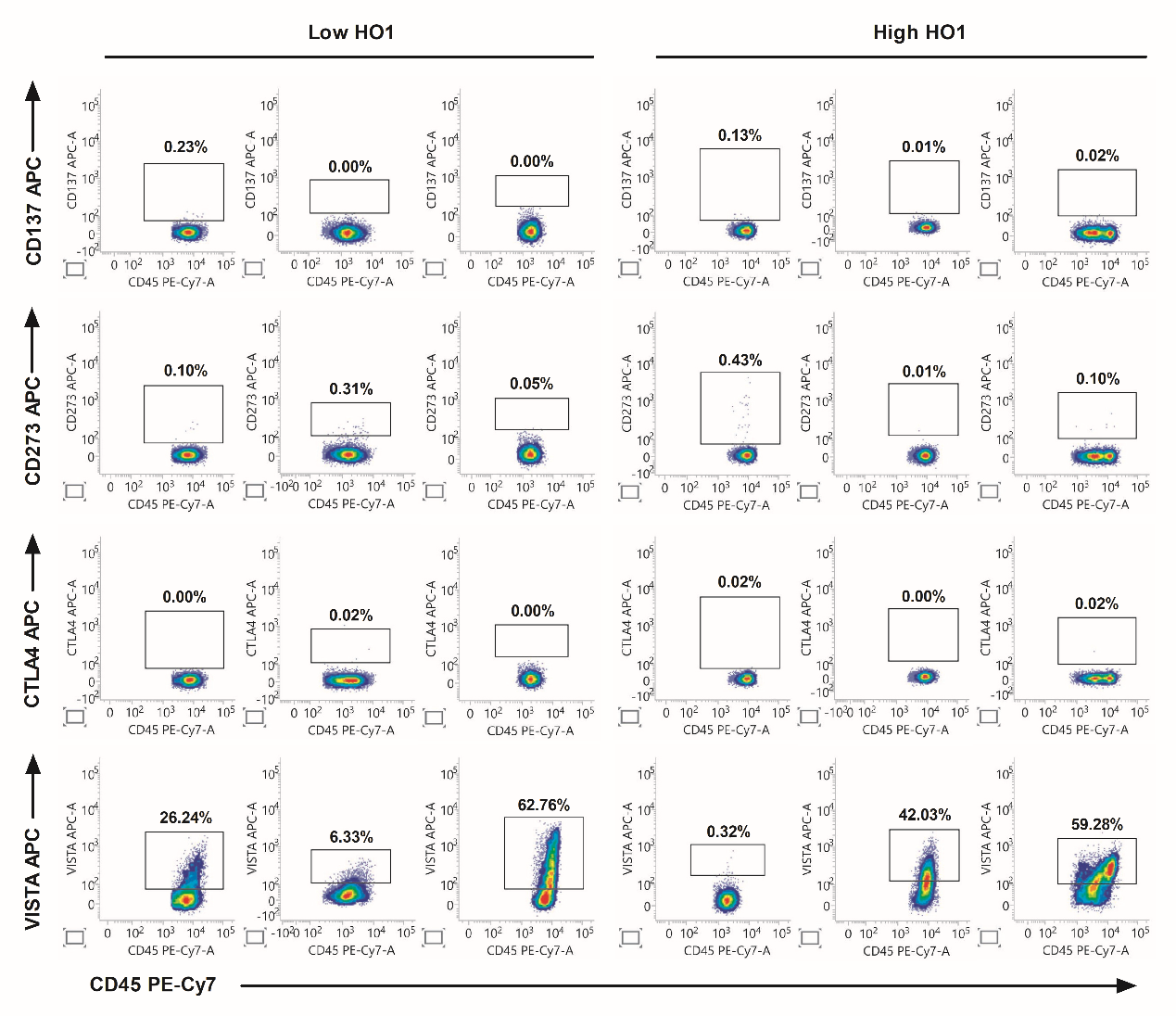


**Fig S3. Correlation between HO1 and immune checkpoint/ligand molecules.**

CD137, CD273, CTLA4, and VISTA expression levels in HO1 high/low AML specimens as determined by flow cytometry.


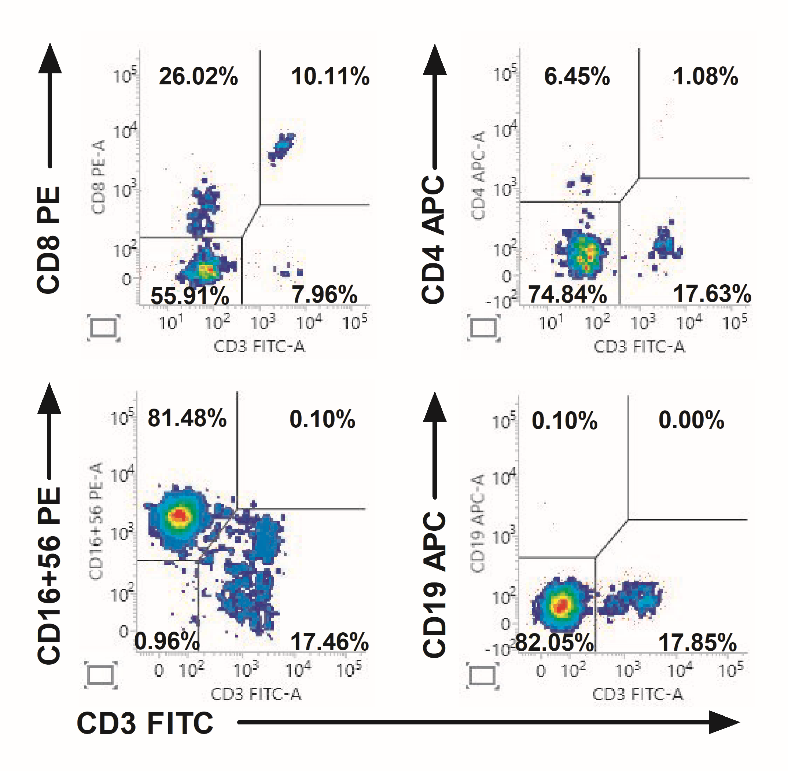


**Fig S4. Immune cell distributions (CD4+ T, CD8+ T, B, and NK cells) in purified primary NK cells.**

Percentage of CD4+ T cells (CD3+ CD4+), CD8+ T cells (CD3+ CD8+), B cells (CD3− CD19+) and NK cells (CD3− CD16+ CD56+) in purified primary NK cells as determined by the TBNK assay.


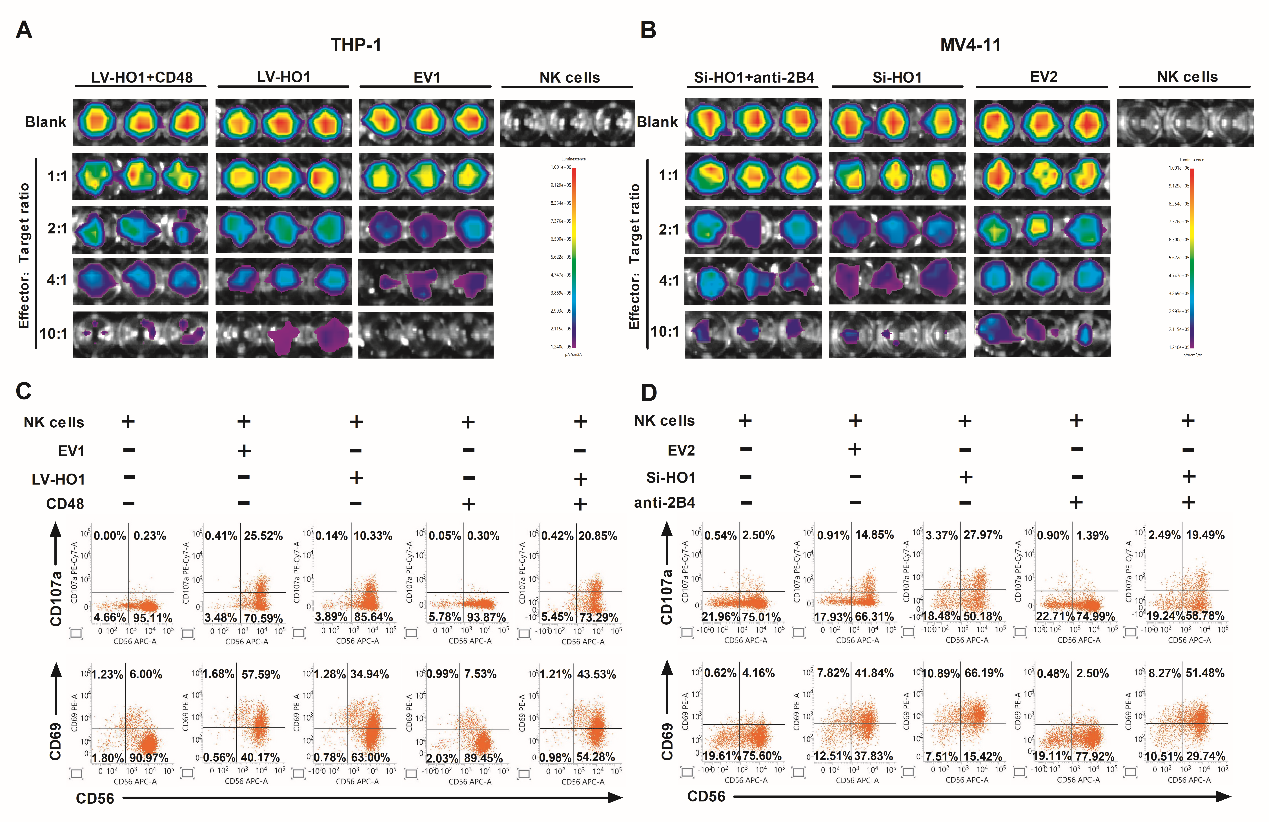


**Fig S5.** **Overexpression of HO1 in AML cells inhibited NK cell cytotoxicity via targeting the CD48-2B4 axis.**

(**A**) Percentage survival of Luc‐labeled transduced THP-1 cells after 2 h co-culture with NK cells in the presence or absence of CD48 protein, as determined by the bioluminescent imaging system. (**B**) Percentage survival of Luc‐labeled transduced MV4-11 cells after 2 h co-culture with NK cells in the presence or absence of anti-2B4 antibody, as determined by the bioluminescent imaging system. (**C**) CD107a or CD69 expression in NK cells after 6 h co-culture of NK cells with transduced THP-1 cells (1:1 E: T ratio) in the presence or absence of CD48 protein. (**D**) Expression of CD107a or CD69 in NK cells after 6 h co-culture of NK cells with transduced MV4-11 cells (1:1 E: T ratio) in the presence or absence of anti-2B4 antibody.


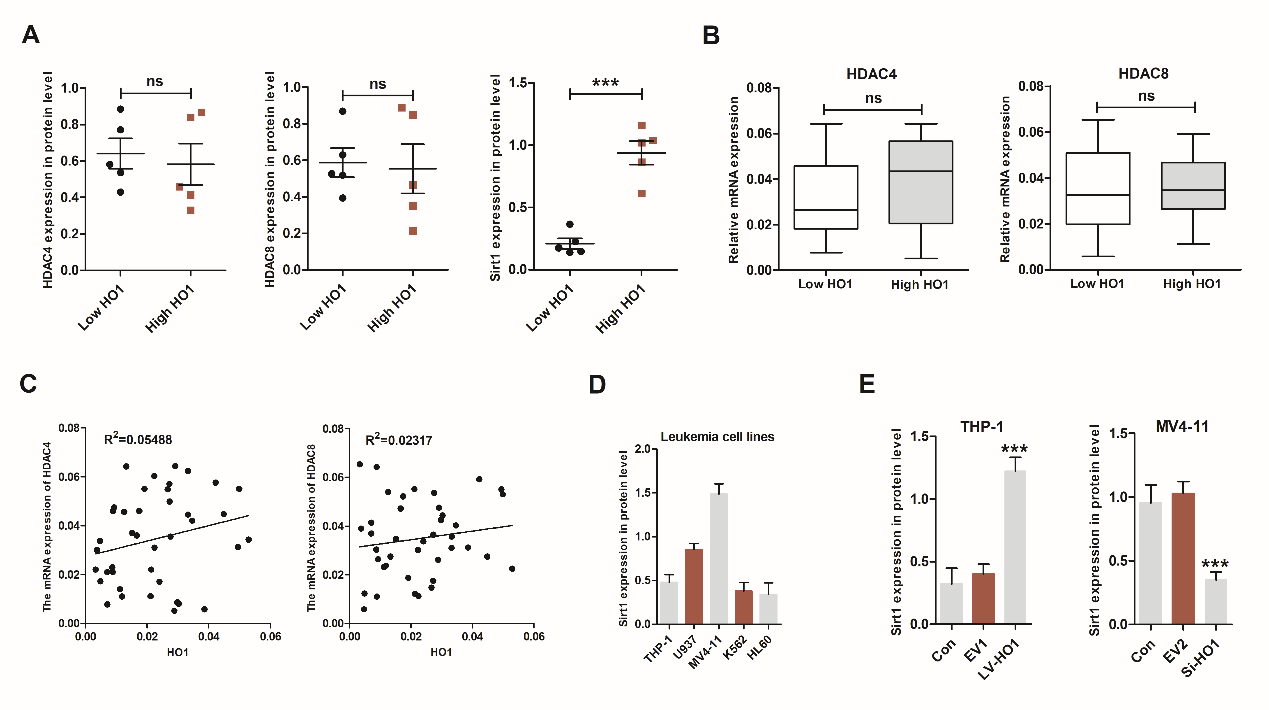


**Fig S6. Correlation between HO1 and HDACs expression.**

(**A**) Quantification of HDAC4, HDAC8, Sirt1 expression in AML samples. (**B**) Expression levels of HDAC4 and HDAC8 genes in the HO1 high/low expressed group as determined by qRT-PCR analysis (n=40). (**C**) Correlation between HO1 and HDAC4 or HDAC8 expression in AML samples as determined by qRT-PCR. (**D**) Sirt1 protein levels in leukemia cell lines as evaluated by western blotting. The relative gray values were shown in histogram. (**E**) The protein level of Sirt1 was detected in THP-1 cells overexpressed HO1 and MV4-11 cells silenced HO1. The relative gray values were shown in histogram. Statistical difference was determined using the Student's t-test. ***p<0.001. ns, no significance.

**
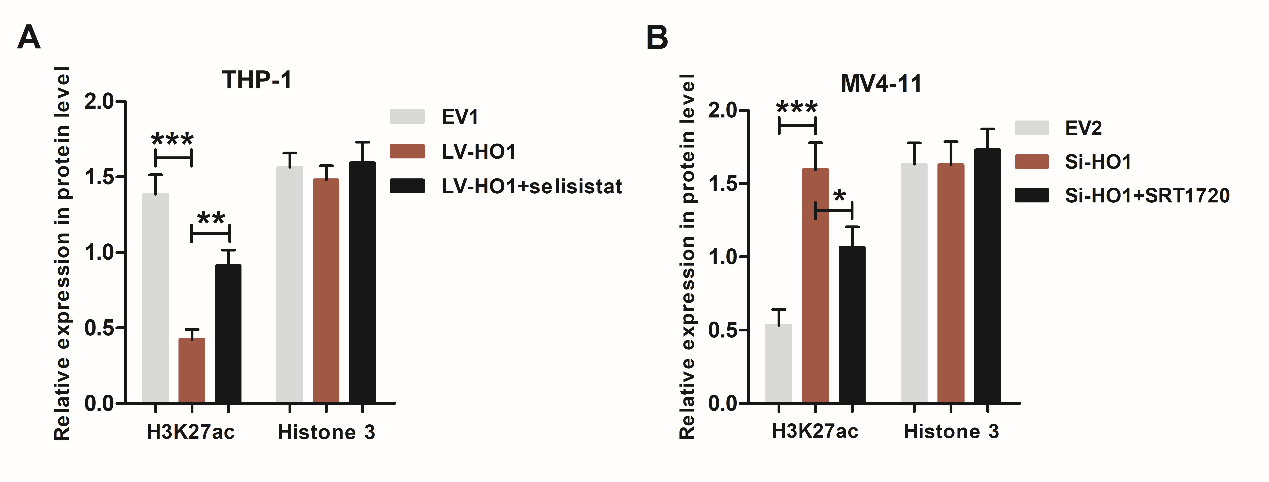
**

**Fig S7.** **H3K27ac mediated the effects of HO1 and Sirt1 on CD48 expression.**

(**A**) H3K27ac levels in EV1 and LV-HO1 THP-1 cells were quantified by western blotting analysis. LV-HO1 cells treated with selisistat are also shown in this graph. The relative gray values were shown in histogram. (**B**) H3K27ac levels in EV2 and Si-HO1 MV4-11 cells were quantified by western blotting analysis. Si-HO1 cells treated with SRT1720 are also shown in this graph. The relative gray values were shown in histogram. Statistical differences were determined using the Student's t-test.. *p<0.05, **p<0.01, ***p<0.001.

**
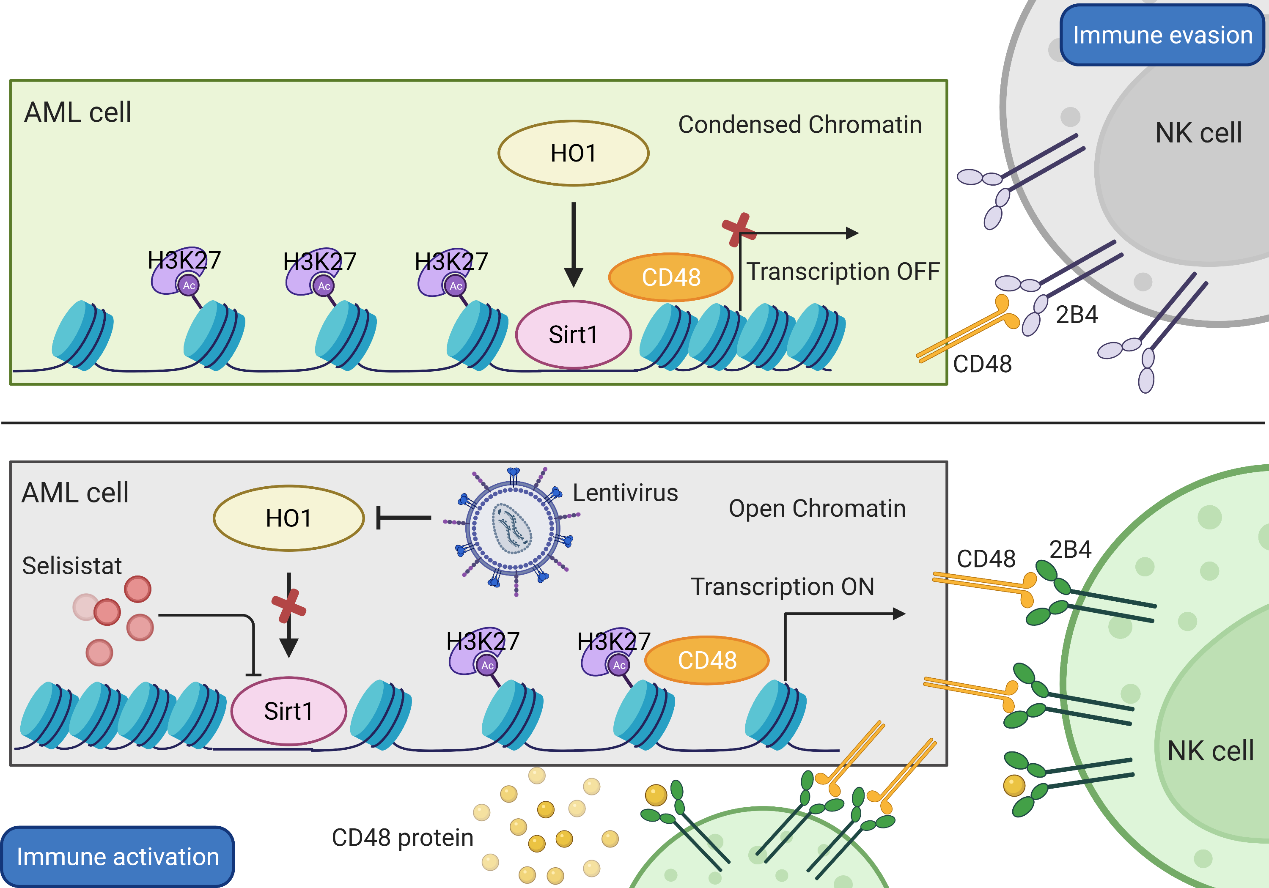
**

**Fig S8.** **Schematic representation of HO1 mediated immune evasion to NK cells in AML.**

In AML cells, HO1 specifically downregulates CD48 levels, a ligand of the NK cell- activating receptor 2B4, thus decreasing the cytotoxic effect of NK cells. Mechanistic studies established that HO1 directly interacted with Sirt1 and increased its expression and the deacetylase activity. Overexpression of HO1 increased Sirt1 in AML cells enabling histone H3K27 deacetylation to suppress CD48 transcription and expression. Administration of selisistat could restore the expression of CD48.

**Additional tables**

**Table S1. Characteristics of patient samples.**

| Samples | Age | Sex | FAB subtype | Disease status | WBC (10^9^L) | Hb (g/l) | PLT (10^9^L) | %Blasts (BM) |
| --- | --- | --- | --- | --- | --- | --- | --- | --- |
| AML1 | 72 | M | M2  M2  M2 | New diagnosis | 88.57 | 52 | 77 | 69.75 |
| AML2 | 21 | M |  | Relapse | 2.04 | 82 | 50 | 38.74 |
| AML3 | 33 | F |  | New diagnosis | 2.20 | 108 | 80 | 29.50 |
| AML4 | 59 | F | M4 | New diagnosis | 272.71 | 57 | 31 | 75.61 |
| AML5 | 57 | M | M4 | New diagnosis | 1.25 | 53 | 13 | 23.60 |
| AML6 | 46 | F | M2 | Relapse | 25.47 | 89 | 41 | 59.04 |
| AML7 | 55 | M | M5 | New diagnosis | 0.83 | 72 | 141 | 57.28 |
| AML8 | 55 | F | M2 | New diagnosis | 19.98 | 48 | 10 | 22.92 |
| AML9 | 57 | M | M5 | Relapse | 411.32 | 53 | 76 | 89.26 |
| AML10 | 26 | M | M4 | Relapse | 6.87 | 148 | 120 | 34.26 |
| AML11 | 52 | F | M2 | New diagnosis | 1.02 | 72 | 10 | 63.46 |
| AML12 | 33 | M | M2 | Relapse | 37.46 | 72 | 12 | 42.75 |
| AML13 | 25 | M | M4 | New diagnosis | 6.50 | 54 | 13 | 26.14 |
| AML14 | 49 | F | M5 | Relapse | 39.77 | 50 | 52 | 23.54 |
| AML15 | 52 | F | M4 | New diagnosis | 9.81 | 71 | 6 | 36.43 |
| AML16 | 44 | F | M2 | Relapse | 0.61 | 69 | 30 | 40.96 |
| AML17 | 77 | M | M2 | New diagnosis | 2.88 | 69 | 64 | 36.24 |
| AML18 | 59 | F | M5 | Relapse | 3.17 | 66 | 22 | 71.65 |
| AML19 | 31 | M | M4 | Relapse | 6.83 | 129 | 35 | 37.01 |
| AML20 | 60 | M | M2 | Relapse | 57.05 | 49 | 26 | 85.51 |
| AML21 | 25 | M | M2 | New diagnosis | 83.74 | 66 | 12 | 40.71 |
| AML22 | 68 | M | M5 | New diagnosis | 134.62 | 85 | 45 | 81.96 |
| AML23 | 33 | M | M5 | Relapse | 45.69 | 157 | 151 | 50.87 |
| AML24 | 37 | M | M5 | New diagnosis | 77.56 | 155 | 89 | 79.22 |
| AML25 | 47 | F | M2 | New diagnosis | 3.08 | 59 | 12 | 77.66 |
| AML26 | 57 | F | M2 | Relapse | 177.51 | 55 | 54 | 42.60 |
| AML27 | 52 | M | M4 | Relapse | 158.38 | 87 | 114 | 81.50 |
| AML28 | 71 | M | M2 | Relapse | 37.07 | 71 | 32 | 90.27 |
| AML29 | 68 | M | M2 | New diagnosis | 0.96 | 85 | 25 | 35.10 |
| AML30 | 53 | M | M5 | New diagnosis | 1.36 | 59 | 112 | 79.01 |
| AML31 | 48 | F | M5 | Relapse | 106.56 | 109 | 110 | 84.88 |
| AML32 | 18 | F | M4 | Relapse | 2.90 | 82 | 54 | 58.73 |
| AML33 | 26 | M | M2 | New diagnosis | 1.33 | 58 | 18 | 53.16 |
| AML34 | 81 | F | M4 | New diagnosis | 1.63 | 102 | 45 | 61.16 |
| AML35 | 34 | M | M5 | Relapse | 151.73 | 56 | 27 | 73.68 |
| AML36 | 88 | M | M5 | Relapse | 2.65 | 66 | 78 | 59.95 |
| AML37 | 34 | F | M5 | New diagnosis | 239.30 | 79 | 151 | 27.14 |
| AML38 | 51 | F | M2 | Relapse | 64.11 | 52 | 32 | 61.10 |
| AML39 | 57 | M | M2 | Relapse | 10.47 | 78 | 24 | 48.11 |
| AML40 | 72 | F | M4 | New diagnosis | 3.80 | 86 | 303 | 28.20 |

Abbreviation: M: male; F: female; AML: acute myeloid leukemia; WBC: white blood cell; Hb: hemoglobin; PLT: platelets; BM: bone marrow.

**Table S2. Antibodies used for flow cytometry.**

| Marker | Fluorochrome | Source | Identifier |
| --- | --- | --- | --- |
| HO1 | FITC | Abcam | Cat#ab69545-100ug |
| CD117 | APC | Beckman Coulter | Cat# B36300 |
| CD33 | PE | Beckman Coulter | Cat# A07775 |
| CD45 | PE-Cy7 | Beckman Coulter | Cat# IM3548 |
| CD45 | PerCP | BD Pharmingen | Cat# 664934 |
| 2B4 | APC-Cy7 | Biolegend | Cat# 329518 |
| CD56 | APC | Beckman Coulter | Cat# IM2474 |
| CD48 | APC | Biolegend | Cat# 336714 |
| CD48 | PE | Biolegend | Cat# 336708 |
| CD366 | APC | BD Pharmingen | Cat# 565558 |
| CD86 | APC | Biolegend | Cat# 374208 |
| CD200R | APC | Biolegend | Cat# 329308 |
| CD30 | APC | Biolegend | Cat# 333909 |
| CD40 | APC | Biolegend | Cat# 334309 |
| CD137 | APC | Biolegend | Cat# 309810 |
| CD273 | APC | BD Pharmingen | Cat# 557926 |
| CTLA4 | APC | Biolegend | Cat# 369611 |
| VISTA | APC | eBioscience | Cat# 17-1088-41 |
| CD107a | PE-Cy7 | Biolegend | Cat# 328618 |
| CD69 | PE | BD Pharmingen | Cat# 555531 |
| CD3/CD8/CD45/CD4 | FITC/PE/PerCP/APC | Agilent | Cat# 8930008 |
| CD3/CD16+CD56/CD45/CD19 | FITC/PE/PerCP/APC | Agilent | Cat# 8930250 |
| Mouse IgG1 isotype control | APC | Beckman Coulter | Cat# IM2475 |
| Mouse IgG1 isotype control | PE | Beckman Coulter | Cat# A07796 |
| Mouse IgG1 isotype control | FITC | Beckman Coulter | Cat# A07795 |
| Mouse IgG1 isotype control | APC-Cy7 | Biolegend | Cat# 409314 |
| Mouse IgG1 isotype control | PE-Cy7 | Biolegend | Cat# 400126 |

**Table S3.** **The characteristics of the primers used for qRT-PCR.**

| GENE | Sequence (5′->3′) | Sequence (5′->3′) |
| --- | --- | --- |
| β-actin | Forward Primer | GAGACCTTCAACACCCCAGC |
|  | Reverse Primer | ATGTCACGCACGATTTCCC |
| HO1 | Forward Primer | ACCCATGACACCAAGGACCAGA |
|  | Reverse Primer | GTGTAAGGACCCATCGGAGAAGC |
| HDAC4 | Forward Primer | GGCCCACCGGAATCTGAAC |
|  | Reverse Primer | GAACTCTGGTCAAGGGAACTG |
| HDAC8 | Forward Primer | TCGCTGGTCCCGGTTTATATC |
|  | Reverse Primer | TACTGGCCCGTTTGGGGAT |
| Sirt1 | Forward Primer | TAGCCTTGTCAGATAAGGAAGGA |
|  | Reverse Primer | ACAGCTTCACAGTCAACTTTGT |
| CD48 | Forward Primer | GGCAGGGTCAGACTTGATCC |
|  | Reverse Primer | GTAGGTGCTGTTGTCCTCTTTC |

**Additional references**

1. Sturm G, Finotello F, Petitprez F, Zhang JD, Baumbach J, Fridman WH, List M, Aneichyk T. Comprehensive evaluation of transcriptome-based cell-type quantification methods for immuno-oncology. Bioinformatics (Oxford, England). 2019;35(14):i436-i45.

2. Li T, Fan J, Wang B, Traugh N, Chen Q, Liu JS, Li B, Liu XS. TIMER: A Web Server for Comprehensive Analysis of Tumor-Infiltrating Immune Cells. Cancer Res. 2017;77(21):e108-e10.

3. Zhang T, Ma D, Wei D, Lu T, Yu K, Zhang Z, Wang W, Fang Q, Wang J. CUDC-101 overcomes arsenic trioxide resistance via caspase-dependent promyelocytic leukemia-retinoic acid receptor alpha degradation in acute promyelocytic leukemia. Anticancer Drugs. 2020;31(2):158-68.
